# High‐Strength and High‐Temperature‐Resistant Structural Battery Integrated Composites via Polymeric Bi‐Continuous Electrolyte Engineering

**DOI:** 10.1002/advs.202407156

**Published:** 2024-10-30

**Authors:** Lijiao Xun, Chen Li, Qinghai Meng, Zilong Wang, Ying Guo, Kun Zheng, Heng Zhou, Tong Zhao

**Affiliations:** ^1^ Key Laboratory of Science and Technology on High‐tech Polymer Materials Institute of Chemistry Chinese Academy of Sciences Beijing 100190 China; ^2^ University of Chinese Academy of Sciences Beijing 100049 China; ^3^ CAS Key Laboratory of Molecular Nanostructure and Nanotechnology CAS Research/Education Center for Excellence in Molecular Sciences Institute of Chemistry Chinese Academy of Sciences Beijing 100190 China

**Keywords:** flame retardance, high‐temperature resistant, phthalonitrile resins, structural battery integrated composites, structure electrolytes

## Abstract

Structural battery integrated composites (SBICs) combining outstanding strength and heat resistance are highly desirable candidates for next generation high speed aircraft. Here, a novel high‐temperature‐resistant bi‐continuous electrolyte based on phthalonitrile resin is presented, allowing the construction of SBICs capable of stable operation across a wide temperature range. Excellent mechanical strength and high ionic conductivity can coexist in a bi‐continuous structure electrolyte (PL_50_) where the phthalonitrile resin serves as the matrix phase and the ionic liquid electrolyte serves as the conductive phase. Benefiting from the thermal stability of the phthalonitrile resin, SBICs assembled with a PL_50_ bi‐continuous electrolyte deliver excellent mechanical performance even at temperatures exceeding 200 °C, with a flexural strength of 299 MPa and a flexural modulus of 31.8 GPa. Additionally, with an increase in operating temperature, PL_50_@SBICs demonstrated enhanced rate performance while maintaining good cycling stability. The demonstration of resisting mechanical abuse at high temperatures and flame retardance further suggests the promise of SBICs with PL_50_ bi‐continuous electrolytes operating under extreme conditions.

## Graphical abstract

The thermal stability of phthalonitrile resin ensures that the bi‐continuous structure electrolyte maintains its structural integrity, excellent mechanical strength, and high ionic conductivity even at extreme temperatures exceeding 200 °C. Consequently, structural battery‐integrated composites assembled with this electrolyte achieve excellent mechanical performance, stable electrochemical properties, and overall safety across a wide temperature range.

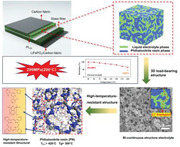

## 1 Introduction

Structural battery integrated composites (SBICs), which integrate mechanical load‐bearing properties with energy storage functionalities, represent a promising approach for lightweight energy storage technologies such as aircraft and electric vehicles, but the relatively poor stability in high‐temperature environments hinders their practical application.^[^ ^1^, ^2^, ^3^, ^4^, ^5^, ^6^, ^7^ ^]^ As a crucial component in SBICs, structural electrolytes typically serve as the matrix that bonds the electrode/reinforcement materials (e.g., carbon fabrics) together, allowing for both mechanical load transfer and lithium‐ion transport.^[^ ^8^, ^9^, ^10^, ^11^, ^12^ ^]^ Recent studies have revealed the remarkable potential of resin materials for developing high‐performance structure electrolytes due to their high mechanical strength, excellent processability, and extensive molecular tunability.^[^ ^13^, ^14^, ^15^, ^16^ ^]^ For example, structure electrolytes with bi‐continuous structures designed using thermosetting resins such as epoxy or cyanate resin as matrix phases effectively balance the traditionally conflicting factors of mechanical strength and ion conductivity, thereby enhancing the overall functionality of SBICs.^[^ ^17^, ^18^ ^]^ However, the thermal decomposition temperature and glass transition temperatures (*T_g_*) of resin materials currently used in structure electrolytes are typically low. As a result, they tend to degrade or soften in harsh environments with operational temperatures typically up to 200 °C or higher, such as the working conditions of high‐speed aircraft during supersonic flight. This degradation leads to interlayer debonding or overall deformation, which is the primary cause of the reduced performance and safety of SBICs in high‐temperature environments.^[^ ^19^, ^20^, ^21^, ^22^ ^]^ Therefore, improving the thermal stability of the resin matrix is crucial for ensuring that structure electrolytes and the SBICs assembled from them maintain their mechanical strength and energy storage efficiency at high temperatures.

Phthalonitrile (PN) resin exhibits excellent mechanical strength, thermal stability, and flame retardance, making it a high‐performance polymer material for extreme environmental applications.^[^ ^23^, ^24^, ^25^, ^26^, ^27^, ^28^ ^]^ During the monomer curing process, the nitrile groups on the benzene ring can undergo intramolecular or intermolecular addition reactions, leading to the formation of heterocyclic crosslinked structures with strong rigidity and high thermal stability, such as isoindoline, phthalocyanine, and triazine.^[^ ^23^, ^29^, ^30^, ^31^ ^]^ As a result, the *T_g_* of PN resins can exceed 350 °C, while the temperature at which 5% of the material's initial mass is lost during thermogravimetric analysis (*T_5%_*) can surpass 450 °C. This cross‐linking structure with high aromatic character also exhibits very high chemical stability, making PN resins resistant to erosion and corrosion by chemical substances, especially oxygen.^[^ ^32^, ^33^, ^34^ ^]^ Moreover, owing to the stable molecular chain structure of PN resin, it maintains structural integrity and stability even at elevated temperatures, ensuring excellent performance retention, including flexural and tensile strength.^[^ ^35^, ^36^, ^37^, ^38^, ^39^ ^]^ Therefore, the synthesis of structure electrolytes using PN resins as the matrix phase undoubtedly provides not only new opportunities to satisfy the requirements of high‐temperature SBICs but also an ideal platform for a comprehensive understanding of the underlying correlations among the structure, mechanics, and energy storage of electrolytes in high‐temperature environments.

In this work, we present a novel high‐temperature‐resistant bi‐continuous electrolyte (PL_50_), utilizing PN resin as the matrix phase and an ionic liquid electrolyte (ILE) comprising bis(trifluoromethane)sulfonamide lithium (LiTFSI) dissolved in 1‐ethyl‐3‐methylimidazolium bis(trifluoromethanesulfonyl)imide (EMIM‐TFSI) ionic liquid as the conductive phase. The formation of a bi‐continuous microstructure ensures that the PL_50_ bi‐continuous electrolyte can provide excellent mechanical strength and high ionic conductivity simultaneously. Moreover, the good thermal stability of the matrix phase enables the PL_50_ to maintain its performance well over a wide temperature range from 25 to 200 °C. Building upon this, we have developed high‐temperature SBICs for the first time, where PL_50_ bi‐continuous electrolyte serves as the polymer matrix, glass fibers act as separators, and carbon fabric and carbon fabric coated with LiFePO_4_ as the anode and cathode, respectively. PL_50_@SBICs can provide excellent mechanical performance at temperatures exceeding 200 °C, with a flexural strength of 299.1 MPa, a flexural modulus of 31.9 GPa, and aging resistance for more than 1000 h. Compared to those at room temperature, PL_50_@SBICs at high temperatures exhibit greater rate performance and can achieve stable cycling in the temperature range of 25–120 °C. Furthermore, even after cutting and drilling at 120 °C or exposure to flame ignition, they can continue to provide energy storage capability, demonstrating the overall safety of high‐temperature PL_50_@SBICs.

## 2 Results and Discussion

**Figure 1:**
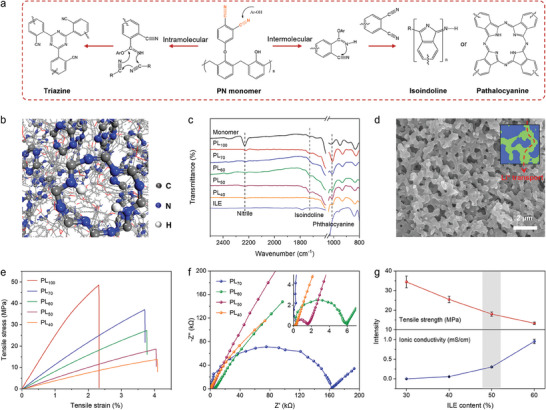
a) The reaction mechanism for phthalonitrile polymerization. b) Crosslinking structure model of the cured PN resin. c) FTIR spectra of the PN resin monomer, ILE, and different bi‐continuous electrolytes. d) SEM image of PL_50_. The scale bar is 2 µm. The inset in (d) is a schematic diagram of lithium‐ion transport pathway in bi‐continuous electrolyte. e) Tensile stress–strain curves of different bi‐continuous electrolytes. f) Impedance spectra of different bi‐continuous electrolytes. The inset in (f) is the magnified image. g) Tensile strength (top) and ionic conductivity (bottom) of the bi‐continuous electrolyte with different ILE contents.

Phthalonitrile (PN) resin was employed as the matrix phase to provide mechanical load‐bearing properties and high‐temperature resistance for the structure electrolyte. As shown in **Figure** **1**a, the nitrile groups on the monomer can undergo intra‐/intermolecular addition reactions under the autocatalysis of the hydroxyl group to form rigid groups, including isoindoline, phthalocyanine, and triazine groups. The cross‐linking structure model for phthalonitrile polymerization by molecular dynamics demonstrated the high‐density distribution of the rigid structures in the highly cross‐linked network, thereby imparting PN resin with excellent thermal stability (Figure 1b; Figure S1, Supporting Information). To ensure the excellent conductivity of the synthesized PN resin‐based structure electrolyte, we selected the LiTFSI/EMIM‐TFSI ionic liquid electrolyte (ILE) as the conducting phase (Figure S2, Supporting Information). Notably, the EMIM‐TFSI solvent has a low vapor pressure, excellent thermal stability, and flame resistance, thereby ensuring the stable performance and safety of the structure electrolyte and subsequent assembled SBICs under high‐temperature operating conditions.^[^ ^40^ ^]^ In the experiment, different masses of the ILE were uniformly mixed with the resin monomer, followed by curing at the optimal curing temperature determined by differential scanning calorimetry (Figure S3, Supporting Information). The mass ratios of the resin monomer to the ILE were 100:0, 70:30, 60:40, 50:50, and 40:60, and the corresponding cured products were denoted as PL_100_, PL_70_, PL_60_, PL_50_, and PL_40_, respectively.

Fourier transform infrared spectroscopy (FTIR) was conducted to confirm the successful synthesis of the PN resin. As shown in Figure 1c, the vibration peak of the nitrile group at 2230 cm^−1^ attributed to the resin monomer is almost absent after sample curing, accompanied by the appearance of isoindoline peaks at 1720 cm^−1^ and phthalocyanine peaks at 1010 cm^−1^.^[^ ^41^, ^42^ ^]^ This indicates that the incorporation of the ILE does not affect the curing reaction of the resin monomer. To observe the microscopic morphology of the structure electrolyte, the ILE in the sample was removed by ethanol extraction. As shown in Figure S4 (Supporting Information), some isolated pores were observed in the resin matrix when the ILE content was 30 wt.%. As the ILE content increased (>50 wt.%), a scanning electron microscopy (SEM) image of the sample revealed the presence of uniform 3D interconnected phases across large areas, confirming the formation of the bi‐continuous structure (Figure 1d). Energy‐dispersive X‐ray spectroscopy mappings further validated that the skeleton in the bi‐continuous structure is composed of PN resin, while the pores are filled with ILE (Figure S5, Supporting Information). The bi‐continuous structure facilitates efficient ion transport and diffusion within the resin matrix, thereby improving the conductivity of the structure electrolyte. The pore distribution and porosity of the bi‐continuous electrolytes were further measured using mercury intrusion porosimetry. As shown in Figure S6 (Supporting Information), the synthesized bi‐continuous electrolytes exhibit a highly uniform pore distribution, with the porosity increasing from 23% to 53% and the pore size increasing from 10 to 2000 nm as the content of the ILE increases (Table S1, Supporting Information).

From the tensile stress–strain curves in Figure 1e and Figure S7 (Supporting Information), it can be observed that the PL_100_ sample exhibits excellent mechanical strength, with a tensile modulus and strength of 2.3 GPa and 40 MPa, respectively. As the ILE content increases, the mechanical strength of the bi‐continuous electrolyte decreases due to the formation of a bi‐continuous structure, which reduces the continuity of the resin phase, as evidenced by the increase in pore size observed in the SEM images. However, the addition of the ILE further enhances the toughness of the bi‐continuous electrolytes, with the elongation at break increasing from 2.3% for the PL_100_ to 4.1% for the PL_50_. Contrary to the trend in mechanical strength, the formation of a bi‐continuous structure favors the construction of continuous and unobstructed ion transport channels, thereby improving the ionic conductivity of the bi‐continuous electrolyte. As shown by electrochemical impedance spectroscopy (EIS), the ionic conductivity of the bi‐continuous electrolyte increases from 1.0 × 10^−4^ to 0.9554 mS cm^−1^ as the electrolyte content increases from 30 to 60 wt.% (Figure 1f). Based on the assessment of the microstructure, mechanical strength, and ionic conductivity, it was determined that a 50 wt.% ILE content achieved optimal comprehensive performance for the PN resin‐based bi‐continuous electrolyte (Figure 1g). Notably, the high‐polarity nitrile groups in the resin monomer enhance its miscibility with the ILE, which can broaden the concentration range of lithium salts concentration required to form the bi‐continuous structure, especially at low concentrations (Figure S8, Supporting Information).^[^ ^43^ ^]^ A lower lithium salt concentration also further reduces the viscosity of the ILE, resulting in enhanced ionic conductivity of the bi‐continuous electrolyte. Therefore, when the lithium salt concentration is 1 m, the PL_50_ exhibits excellent comprehensive performance, an ionic conductivity of 0.9879 mS cm^−1^, a tensile modulus of 470 MPa, and a tensile strength of 13.3 MPa (Figure S9 and Table S2, Supporting Information).

**Figure 2:**
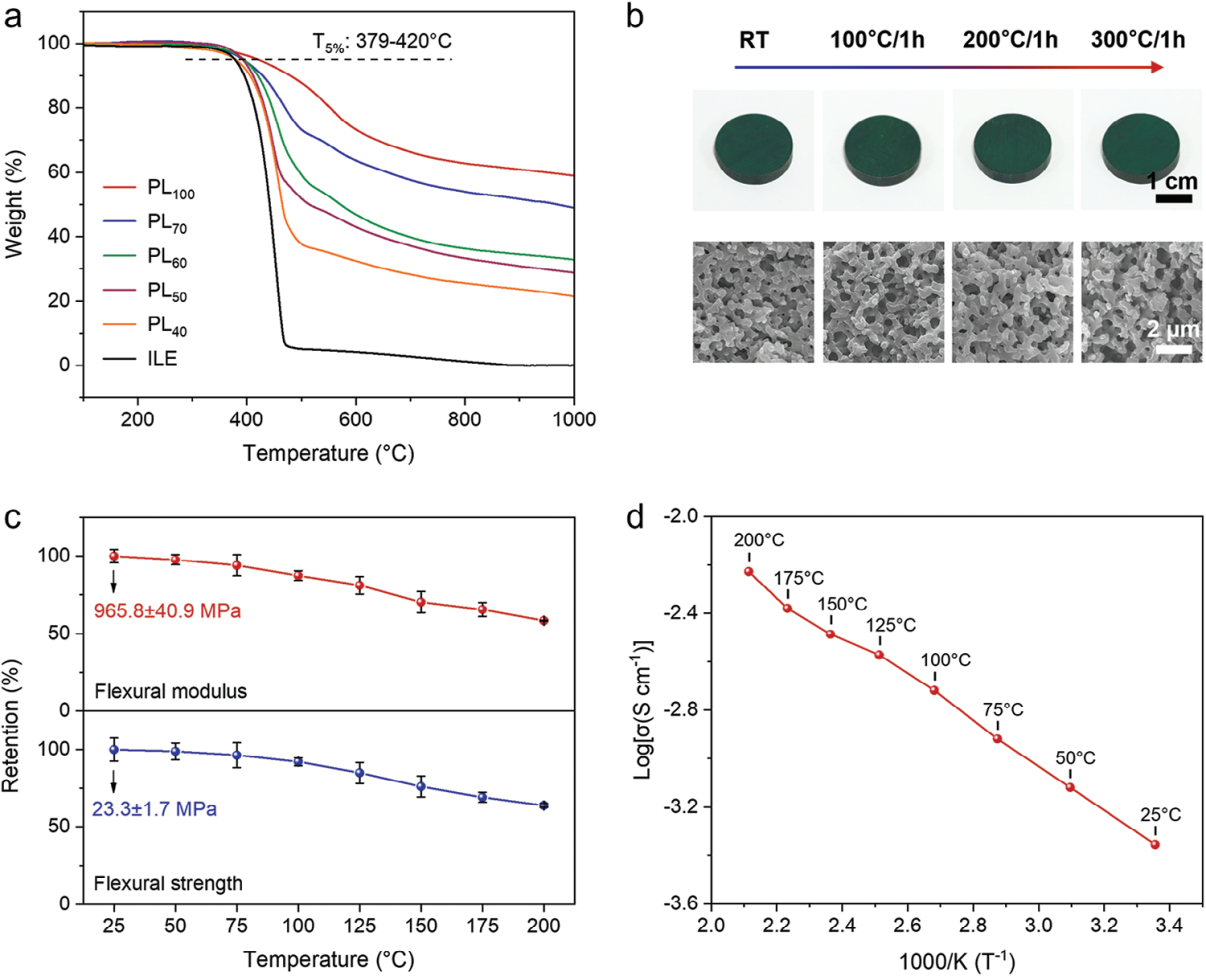
a) TGA curves for different bi‐continuous electrolytes and the ILE. b) Photographs (top) and SEM images (bottom) of the PL_50_ placed at different temperatures for 1 h. Scale bars are 1 cm (top) and 2 µm (bottom). c) Retention plots of the flexural strength and modulus of the PL_50_ at different temperatures. d) EIS plots of the PL_50_ at different temperatures.

The thermal stability of the bi‐continuous electrolytes is a prerequisite for their excellent performance and safety at high temperatures. As shown in the thermogravimetric analysis (TGA) curves in **Figure** **2**a, the pure PN resin (PL_100_) exhibited excellent thermal stability, with a 5% weight loss temperature (*T_5%_*) exceeding 420 °C. Despite the slightly decreased thermal stability of the bi‐continuous electrolyte compared to that of the pure PN resin due to the introduction of the ILE (Figure S10, Supporting Information), the *T_5%_* values for PL_70_, PL_60_, PL_50_, and PL_40_ remain impressive at 394, 391, 387, and 379 °C, respectively. These values significantly surpass the functional temperature range of traditional structural electrolytes. As shown in Figures S11 and S12 (Supporting Information), the bi‐continuous electrolytes exhibit excellent air stability, with no significant changes in their structure, composition, or electrochemical performance even after 30 days of exposure. More importantly, after maintaining the PL_50_ sample at temperatures ranging from 100 to 300 °C for 1 h, no substantial changes were observed in its size or morphology (Figure 2b, top). After heating at 300 °C for 1 h, the thermal expansion rate was only 1%, further demonstrating the excellent thermal stability of the electrolyte (Figure S13, Supporting Information). Additionally, as observed from the corresponding SEM images (Figure 2b, bottom), the bi‐continuous structure showed no noticeable deformation or collapse even at the elevated temperature of 300 °C.

In addition to the excellent thermal stability, we also evaluated the mechanical strength and ionic conductivity of the PL_50_ bi‐continuous electrolytes at high temperatures. As shown in Figure 2c, the PL_50_ exhibited a flexural modulus of 845 MPa and a flexural strength of 21.5 MPa at 100 °C, retaining over 90% of their values compared to room temperature conditions. Even at 200 °C, the PL_50_ samples maintain good mechanical strength, with a flexural modulus of 563 MPa and a flexural strength of 14.9 MPa. Generally, the significant decrease in the mechanical strength of the bi‐continuous electrolytes at high temperatures is mainly attributed to the increased mobility of the ionic liquid, which further weakens the intermolecular forces between polymer chains and leads to chain relaxation. As illustrated by the dynamic thermomechanical analysis curves, both the PN resin and PL_50_ samples exhibit glass transition temperatures (*T_g_*) exceeding 300 °C (Figure S14, Supporting Information). The highly crosslinked polymer network of the resin matrix relaxes slowly below the *T_g_*, resulting in comprehensive high mechanical retention. Moreover, increased temperature reduces the viscosity of the ILE, which helps ion migration and thus increases the ionic conductivity.^[^ ^44^ ^]^ Figure 2d shows the relationship between the ionic conductivity and temperature of the PL_50_, following the Arrhenius equation. At 200 °C, the ionic conductivity is 5.92 mS cm^−1^, indicating the suitability of the electrolyte for this temperature condition. These results show that the PL_50_ is an excellent high‐temperature‐resistant bi‐continuous electrolyte.

**Figure 3:**
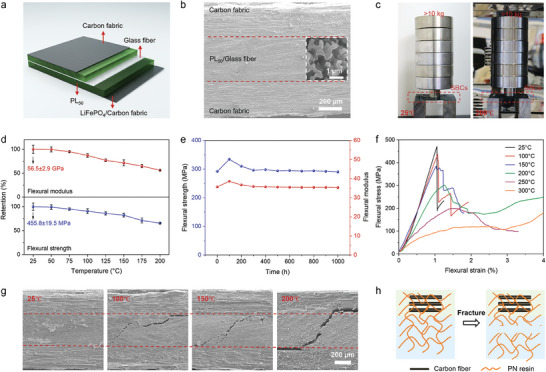
a) Schematic diagram of the device structure of PL_50_@SBICs. b) Cross‐sectional SEM image of PL_50_@SBICs. Scale bar is 200 µm. The inset in (b) is the magnified SEM image of PL_50_. Scale bar is 2 µm. c) Photographs of PL_50_@SBICs supporting more than 10 kg weight at 25 °C (left) and 120 °C (right). d) Retention plots of the flexural strength and modulus of PL_50_@SBICs at different temperatures. e) Flexural strength and modulus of PL_50_@SBICs at 200 °C for different aging durations. f) Flexural stress–strain curves of PL_50_@SBICs at different temperatures. g) Cross‐sectional SEM images of PL_50_@SBICs after flexural testing at different temperatures. Scale bar is 200 µm. h) The schematic diagram of the fracture behavior of PL_50_@SBICs at high temperatures.

To evaluate the performance of SBICs based on the PL_50_ bi‐continuous electrolyte (labeled PL_50_@SBICs), we designed the device structure illustrated in **Figure** **3**a. In this structure, PL_50_ serves as both the structural electrolyte and polymer matrix, with glass fiber (GF) as the separator, and carbon fabric (CF) and CF coated with LiFePO_4_ as the anode and cathode, respectively. To ensure the uniformity and consistency of the PL_50_ bi‐continuous electrolyte in the device, we employed a vacuum bag prepreg molding process to combine the various components into the SBICs (Figure S15, Supporting Information). The rheological curves indicate that the viscosity of the PL_50_ precursor remains relatively low (0.1–0.043 Pa s) within the processing temperature range of 100–200 °C, which facilitates their thorough infiltration and diffusion into both the CF electrodes and GF separators prior to curing (Figure S16, Supporting Information). As a result, as observed in the SEM image of the cross‐section of PL_50_@SBICs, the PL_50_ bi‐continuous electrolyte completely fills the internal spaces of the CF electrodes and GF separators, forming a uniform composite structure without discernible interlayer separation (Figure 3b). Also, the formation of a clear bi‐continuous structure can be observed from the inset in Figure 3b. The excellent interfacial adhesion between the PL_50_ bi‐continuous electrolyte and CF electrodes facilitates the effective transfer of mechanical loads and lithium ions between the layers. As shown in Figure 3c, PL_50_@SBICs has high mechanical strength, showing no noticeable bending when subjected to a load exceeding 10 kg. Even after exposure to a high‐temperature environment at 200 °C, PL_50_@SBICs continued to provide load‐bearing capacity, with no substantial changes in morphology observed.

As shown in Figure 3d, an initial flexural strength of 455.8 MPa and a flexural modulus of 56.5 GPa were obtained for PL_50_@SBICs operated at 25 °C. Furthermore, it has a tensile strength of 1010 MPa and a tensile modulus of 69.04 GPa (Figure S17, Supporting Information). The remarkable mechanical properties of PL_50_@SBICs can be attributed to the synergistic effect of carbon fibers as reinforcements and PL_50_ as the polymer matrix. As expected, due to the excellent thermal stability of the PL_50_ bi‐continuous electrolyte, both the flexural strength and modulus of PL_50_@SBICs were well retained under high‐temperature conditions, with a retention of ≈90% at 100 °C and ≈60% at 200 °C. Notably, the flexural strength and modulus at 200 °C are 299 MPa and 31.8 GPa, respectively, which, to our knowledge, are the highest values reported to date.^[^ ^1^, ^3^, ^15^, ^17^, ^18^, ^45^, ^46^ ^]^ As shown in Figure 3e, the flexural strength and modulus of PL_50_@SBICs did not significantly decrease after continuous heating at 200 °C for 1000 h, indicating good thermal oxidation stability. At temperatures above 300 °C, highly cross‐linked polymer networks of PN resin enable PL50@SBICs to exhibit mechanical retention thus enhancing their safety in extreme environments (Figure S18a, Supporting Information).^[^ ^35^ ^]^ The excellent mechanical performance of PL_50_@SBICs at high temperatures can be further explained by changes in the fracture behavior. As shown in Figure 3f, the stress–strain curves of PL_50_@SBICs before 100 °C are consistent with those of the PL_50_ bi‐continuous electrolyte, and both fracture before yielding, which is a typical brittle fracture behavior. As the temperature increased to 200 °C, PL_50_@SBICs exhibited yielding before fracture, indicating improved toughness. Within the temperature range of 25–200 °C, noticeable electrolyte fracture can be observed in the SEM images of PL_50_@SBICs cross‐sections, in which the resin matrix can still bond the CF well and thus achieve effective load transfer (Figure 3g,h). At operating temperatures above 250 °C, partial delamination of the CF layers begins due to the decrease in the interfacial adhesion between the resin matrix and the CF, which may be the primary reason for the decrease in the mechanical performance (Figure S18b, Supporting Information).

The electrochemical properties of PL_50_ bi‐continuous electrolyte were then investigated. First, the electrochemical stability window of the electrolytes was determined using linear sweep voltammetry at a scan rate of 1 mV s^−1^, as shown in Figure S19 (Supporting Information). Compared to LE (3.84 V), PL_50_ exhibits a higher decomposition voltage (4.84 V), which is attributed to the uncross‐linked nitrile groups in the phthalonitrile resin that are difficult to oxidize.^[^ ^47^, ^48^ ^]^ Moreover, its strong electron‐withdrawing capability can further lower the highest occupied molecular orbital (HOMO) level of the molecule.^[^ ^49^, ^50^ ^]^ As a result, the oxidation potential window of PL_50_ is greatly increased. Additionally, the Li^+^ transference number ($t_{Li^{+}}$) of the electrolyte was measured using chronoamperometry and EIS. The results show that the $t_{Li^{+}}$ value of PL_50_ is 0.20, higher than the 0.17 of ILE (Figure S20, Supporting Information). The increase in $t_{Li^{+}}$ is mainly attributed to the unreacted nitrile groups and the nitrogen atoms in the cross‐linked structure, which attract lithium ions through Lewis acid–base interactions, thereby promoting the dissociation of lithium salts (Figure S21, Supporting Information). Therefore, combined with the excellent thermal stability and improved electrochemical performance of the PL_50_ bi‐continuous electrolyte, PL_50_@SBICs is capable of providing enough energy storage capacity at high temperatures.

**Figure 4:**
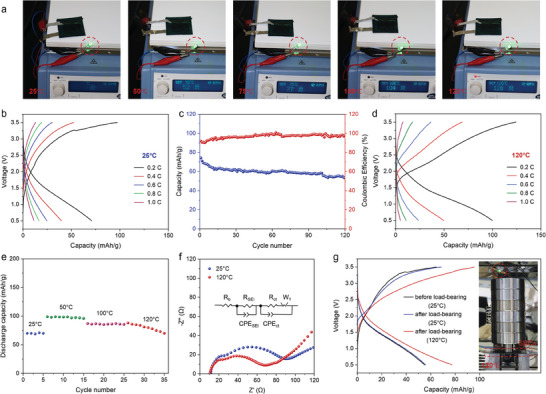
a) Photographs of PL_50_@SBICs operating on a continuous heating flat heater. b) Voltage profiles of PL_50_@SBICs at 25 °C with the different rates. c) Cycling performance of PL_50_@SBICs at a rate of 0.2 C at 25 °C, tested without applying pressure. d) Voltage profiles of PL_50_@SBICs at 120 °C with the different rates. e) Cycling stability of PL_50_@SBICs at 25, 50, 100, and 120 °C, cycled at a rate of 0.2 C. f) EIS plots of PL_50_@SBICs at 25 and 120 °C. The inset in (f) is the equivalent circuit. g) Charge–discharge curves of PL_50_@SBICs before and after supporting more than 10 kg of weight at 120 °C. The inset is a photograph of PL_50_@SBICs continuing to drive LEDs after supporting more than 10 kg of weight at 120 °C.

As shown in **Figure** **4**a, PL_50_@SBICs are able to continuously power the LED as the ambient temperature increases from 25 to 120 °C, with no observed deformation or other faults during the testing process. We further compared the electrochemical performance of PL_50_@SBICs at 25 and 120 °C. It should be noted that, to accurately assess the contribution of the PL_50_ bi‐continuous electrolyte to the electrochemical performance of SBICs at high temperatures, we did not load any other high‐capacity active materials on the CF anode. As shown in Figure 4b, a specific capacity of 77 mAh g^−1^ was obtained for PL_50_@SBICs operated at 0.2 C at 25 °C. After 120 cycles at a rate of 0.2 C, PL_50_@SBICs exhibit a retained discharge capacity of 55 mAh g^−1^ and an average Coulombic efficiency above 99% (Figure 4c). After more than 120 cycles, the Coulombic efficiency remains stable, but the specific capacity begins to gradually decline (Figure S22, Supporting Information). This decline is likely due to the poor cycling performance of the unmodified carbon fibers. Notably, the structure and morphology of PL_50_@SBICs remained intact throughout the cycling process (Figure S23, Supporting Information). Given the strong adhesive force between the electrodes in the PL_50_@SBICs, no additional stacking pressure was applied during the aforementioned cycling tests; instead, testing was conducted under standard environmental conditions (Figure S24, Supporting Information). In addition, cycling tests conducted under a load exceeding 10 kg further confirmed the excellent mechanical performance of PL_50_@SBICs (Figure S25, Supporting Information). When the operating temperature increased to 120 °C, PL_50_@SBICs exhibited improved rate performance (Figure 4d). The specific capacities at 0.2 C, 0.4 C, 0.6 C, and 0.8 C were 99.9, 50.0, 23.6, and 10.9 mAh g^−1^, respectively. The specific capacity at 1 C is slightly lower than that at room temperature, which may be due to the side reactions, such as gas production from electrolyte decomposition and solid electrolyte interphase decomposition at the anode, which significantly increase at the electrode interface at high temperatures.^[^ ^51^ ^]^ Therefore, PL_50_@SBICs achieved a broad operating temperature window from room temperature to 120 °C, with good cycling performance demonstrated at each temperature stage (Figure 4e).

As shown in the EIS plot in Figure 4f, the charge transfer of PL_50_@SBICs decreases from 59.15 Ω at 25 °C to 32.30 Ω at 120 °C (Table S3, Supporting Information), which may be one of the reasons for the improved electrochemical performance at high temperatures. The increased ionic conductivity of the bi‐continuous electrolyte at high temperatures leads to an increased lithium‐ion concentration at the electrode, thereby facilitating more effective utilization of the active materials in the cathode. This enhancement is corroborated by the increase in peak current from 0.06 mA mg^−1^ at 25 °C to 0.28 mA mg^−1^ at 120 °C in the cyclic voltammetry curves at a scan rate of 5 mV s^−1^ (Figure S26, Supporting Information). Both improvements can be attributed to the excellent thermal stability and outstanding mechanical strength of the PL_50_ bi‐continuous electrolyte at high temperature. Even at 120 °C, it maintains the integrity of the device structure, including electrode separation, stable interfaces, and a 3D bi‐continuous structure. This effectively reduces the risk of internal short circuits and electrolyte leakage. To further verify the practical application potential of PL_50_@SBICs, we evaluated their comprehensive performance as both a mechanical load‐bearing unit and an energy storage unit (Figure 4g). The charge–discharge curves indicate that PL_50_@SBICs can maintain a better specific capacity before and after bearing the load, even with drastic changes in the external temperature. As shown in the inset of Figure 4g, at 120 °C, PL_50_@SBICs can stably power the LED while supporting a weight of more than 10 kg.

**Figure 5:**
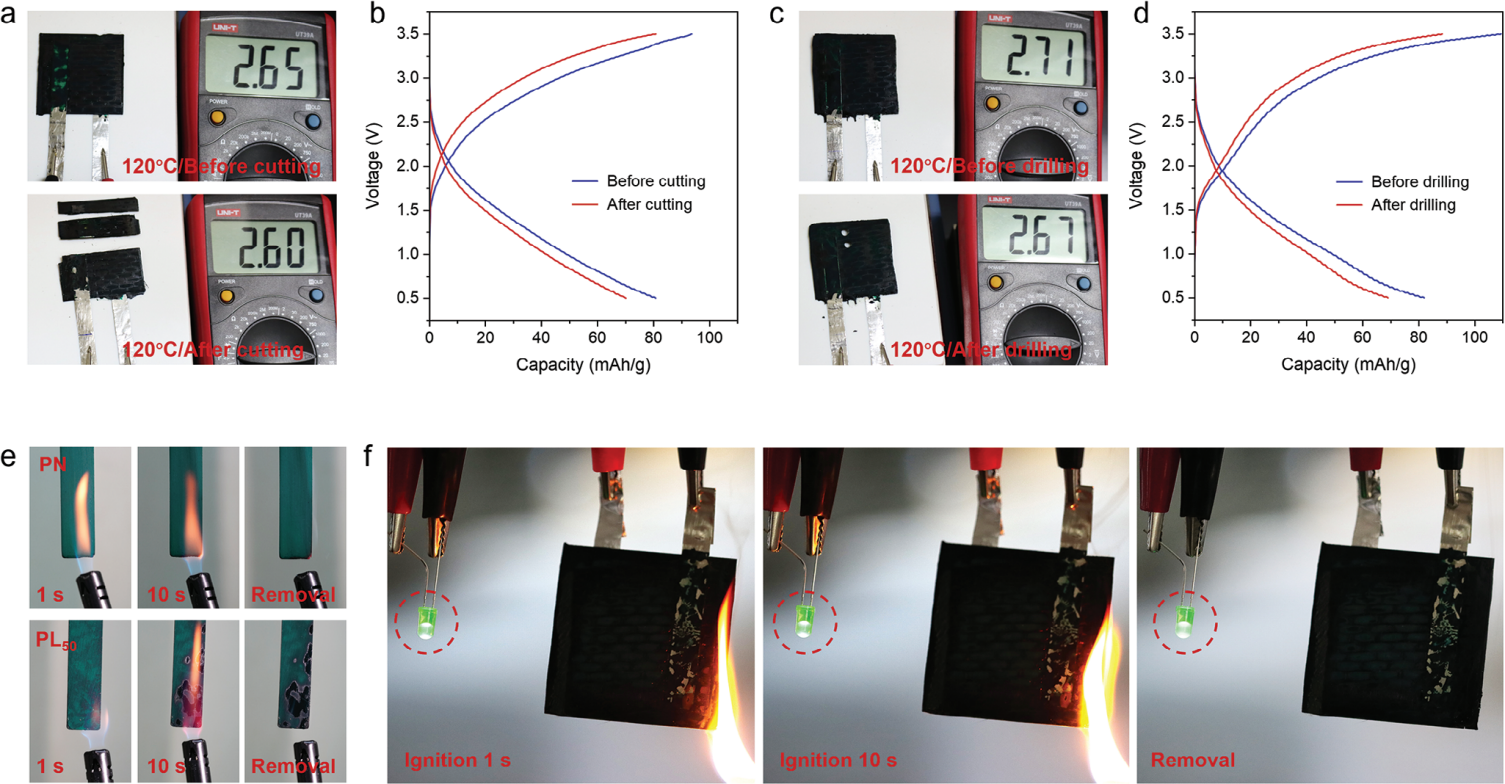
a) Photograph of PL_50_@SBICs continuing to drive LEDs before and after being cut into three pieces on a 120 °C flat heater. b) Charge–discharge curves of PL_50_@SBICs before and after cutting. c) Photograph of PL_50_@SBICs continuing to drive LEDs before and after drilling on a 120 °C flat heater. d) Charge–discharge curves of SBICs before and after drilling. e) Flame tests of PN resin (top) and PL_50_ (bottom). f) Flame tests of the PL_50_@SBICs.

Overall, PL_50_@SBICs work well at a wide range of temperatures, from ambient to high, and demonstrate excellent mechanical properties as well as stable electrochemical performance. Combined with its excellent molding capability, the composite material holds promising prospects for practical applications in high‐temperature SBICs. As shown in Figure S27 (Supporting Information), we modeled the composite material after high‐speed aircraft and tailored it into various shapes, including curved surface, barrel, and conical, to suit the practical application scenarios of high‐temperature structural batteries within them. Notably, the molded PL_50_@SBICs can still maintain a stable output voltage (Figure S28, Supporting Information). It is foreseeable that any minor structural damage to SBICs in high‐temperature environments may pose more severe safety risks, including electrode short circuits, electrolyte leakage and combustion. Therefore, we conducted cutting, drilling, and ignition operations to simulate the harsh conditions encountered in practical applications, further assessing the safety of the PL_50_@SBICs at high temperatures. As shown in **Figure** **5**a, even when the PL_50_@SBICs were cut into three pieces, the remaining part continued to maintain a stable operating voltage at 120 °C. The charge–discharge tests indicate that the PL_50_@SBICs maintain a stable specific capacity after cutting, with no observed short circuits or other faults (Figure 5b). Furthermore, after drilling two holes in the PL_50_@SBICs, they still operate normally at high temperatures (Figure 5c,d). Benefiting from the excellent thermal stability and mechanical strength of the PL_50_ bi‐continuous electrolyte, there was no deformation or breakage at the cut and drill areas, and no leakage of the ionic liquid electrolyte occurred under drastic environmental temperature changes.

Flame retardancy is also an important aspect of ensuring the safe operation of PL_50_@SBICs. As shown in Figure 5e (top), the PN resin samples were directly exposed to a butane flame and did not ignite within 10 s. Similarly, the PL_50_ samples also exhibited excellent flame retardancy; even when there was some leakage of the ionic liquid electrolyte on the surface, there was no ignition (Figure 5e, bottom). Limiting oxygen index (LOI) and vertical burning (UL‐94) tests were further conducted to evaluate the combustibility and flame resistance of the PN resin and PL_50_ in air. The results demonstrate excellent flame retardancy performance for both the PN resin and PL_50_ samples, with LOI values exceeding 32% and a UL‐94 rating of V‐0 (Table S4, Supporting Information). As a result, the PL_50_@SBICs were also endowed with excellent flame retardancy with an LOI of 58.7%. This improvement in LOI can be attributed to the carbon fiber electrodes used in PL_50_@SBIC, which demonstrated excellent flame retardancy (Figure S29, Supporting Information). In flame tests, PL_50_@SBICs remained unignited for 10 s and continued to power the LED (Figure 5f). These results indicate that the PL_50_ bi‐continuous electrolyte will endow SBICs with remarkable performance and high safety at high temperatures, which is crucial for the practical application of SBICs.

## 3 Conclusion

In summary, we have developed a high‐performance bi‐continuous electrolyte suitable for high‐temperature environments based on the PN resin. Through optimization of the ILE content and lithium salt concentration, the PL_50_ electrolyte with a bi‐continuous structure was successfully synthesized, achieving optimal comprehensive performance. Characterization of the mechanical and electrochemical properties at high temperatures revealed that the PL_50_ bi‐continuous electrolytes had good performance retention at 200 °C, with a flexural modulus of 563 MPa, flexural strength of 14.9 MPa, and ionic conductivity of 5.92 mS cm^−1^. Consequently, SBICs constructed with PL_50_ can reliably operate over a wide temperature range of 25–200 °C. At 200 °C, PL_50_@SBICs exhibited excellent mechanical performance, with a flexural strength of 299 MPa, a flexural modulus of 31.8 GPa, and aging resistance for more than 1000 h. Compared to room temperature conditions, PL_50_@ SBICs exhibited enhanced rate performance at 120 °C while maintaining good cycling performance. Additionally, PL_50_@SBICs exhibit resistance to mechanical abuse at high temperatures and flame retardancy. This work not only provides a feasible solution for developing high‐temperature SBICs but also offers new insights into the relationships among structure, mechanics, and energy storage in high‐temperature environments.

## Conflict of Interest

The authors declare no conflict of interest.

## Supporting information



Supporting Information

## Data Availability

The data that support the findings of this study are available from the corresponding author upon reasonable request.
