# Supplementary material for: High‐Strength and High‐Temperature‐Resistant Structural Battery Integrated Composites via Polymeric Bi‐Continuous Electrolyte Engineering
Source: Adv Sci (Weinh). 2024 Oct 30;11(47):2407156. doi: 10.1002/advs.202407156 (PMC11653619; doi:10.1002/advs.202407156)
Supplement: Supplementary file 1 — Supporting Information [file ADVS-11-2407156-s001.docx]

Supporting Information

**High-Strength and High-Temperature-Resistant Structural Battery Integrated Composites via Polymeric Bi-Continuous Electrolyte Engineering**

*Lijiao Xun, Chen Li, Qinghai Meng, Zilong Wang, Ying Guo, Kun Zheng, Heng Zhou* and Tong Zhao**

L. Xun, Dr. Z. Wang, Prof. Y. Guo, Prof. K. Zheng, Prof. H. Zhou, Prof. T. Zhao

Key Laboratory of Science and Technology on High-tech Polymer Materials

Institute of Chemistry Chinese Academy of Sciences

Beijing, 100190, China

E-mail: zhouheng@iccas.ac.cn; tzhao@iccas.ac.cn

L. Xun, C. Li, Prof. H. Zhou, Prof. T. Zhao

University of Chinese Academy of Sciences

Beijing, 100049, China

C. Li, Prof. Q. Meng

CAS Key Laboratory of Molecular Nanostructure and Nanotechnology

CAS Research/Education Center for Excellence in Molecular Sciences

Institute of Chemistry Chinese Academy of Sciences

Beijing, 100190, China

Experimental Methods

*Materials*: phthalonitrile monomer was prepared in our laboratory by a previously reported method^[1]^. 1-Ethyl-3-methylimidazolium bis(trifluoromethanesulfonyl)imide (EMIM-TFSI) was supplied by J&K Scientific. Bis(trifluoromethane) sulfonamide lithium salt (LiTFSI) was supplied by Aladdin. Unidirectional carbon fabric (T-700) was purchased from Toray Industries, NC (Japan). LiFePO_4_, super P, and polyvinylidene fluoride (PVDF) were purchased from Shenzhen Kejing Star Technology Co., Ltd. N-methyl-2-pyrrolidone (NMP, 99.9%) were purchased from Innochem Technology Co., Ltd. Whatman glass microfiber separator (Whatman GF/A, 260 μm thick) supplied by GE healthcare Whatman. All materials without specific mention were used as received.

*Preparation of bi-continuous electrolyte:* The ionic liquid electrolyte was prepared by dissolving LiTFSI in EMIM-TFSI and stirring mechanically at 60°C, where the mass ratio of LiTFSI: EMIM-TFSI was 3:7. The phthalonitrile monomer and ionic liquid electrolyte were evenly mixed by magnetic stirring at 120°C. After all the components were fully uniform, the mixture was put into a vacuum oven for 1h to remove the bubbles and get the bi-continuous electrolyte precursor. The bi-continuous electrolyte precursor mixture was transferred into the mold for curing. The cured process was 175°C for 4 h, 200°C for 4 h, and 250°C for 4 h. The cured samples were cut into discs for subsequent electrochemical testing. Preparation of bi-continuous electrolytes PL_100_, PL_70_, PL_60_, PL_50_ and PL_40_ by changing the different mass ratios of phthalonitrile monomer and ionic liquid electrolyte. Bi-continuous electrolytes with different lithium salt concentrations were obtained by changing the feeding mass ratio of LiTFSI and EMIM-TFSI. The mass ratio of LiTFSI: EMIM-TFSI for PL_50_/1M electrolyte was 3:16.

*Fabrication of PL_50_@SBICs:* LiFePO_4_, Super P and PVDF were dispersed in the solvent NMP at a mass ratio of 80:10:10 to form a uniform slurry. The slurry was uniformly coated on the carbon fabric with a coater and dried in a vacuum at 120°C for 12 h to obtain the LiFePO_4_ cathode with a loading of ~ 2 mg cm^-2^ on carbon fabric. The carbon fabric, glass fiber separator and LiFePO_4_ coated carbon fabric were stacked in this order, and the bi-continuous electrolyte was fully impregnated with each component using a vacuum bag prepreg molding process. Finally, the structural battery integrated composite was obtained after curing the assembled battery. And the cured process was the same as that of the bi-continuous electrolyte.

*Materials Characterizations:* Fourier transform infrared spectroscopy (FT-IR) measurements were conducted on a TENSOR-27 spectrometer. Thermogravimetric analysis (TGA) under nitrogen atmosphere (50 mL·min^-1^) was carried out on an STA 409 PC instrument (NETZSCH Scientific Instruments Trading (Shanghai) Ltd. China) at a heating rate of 10 ^o^C·min^-1^. Differential scanning calorimetry (DSC) analysis was obtained on a DSC822e instrument (METTLER TOLEDO, Switzerland) thermal analyzer at a heating rate of 10 ^o^C·min^-1^ under nitrogen atmosphere. Dynamic mechanic analysis (DMA) tests were performed on DMA 242e (Netzsch, Germany) at a fixed frequency of 1 Hz with three-point bending mode, and the oven was heated from 30°C to 350°C at a heating rate of 5 °C·min^−1^ in a nitrogen atmosphere. The cured samples for DMA were cut into rectangular specimens of dimensions 30 mm × 10 mm × 2 mm. Thermal expansion measurements were conducted using a DIL402 instrument (Netzsch, Germany) with sample dimensions of 25 mm × 5 mm × 5 mm. The microstructure was observed using a S-8020 scanning electron microscope (Hitachi, Japan) operated at 10 kV. To detect the flame retardancy of bi-continuous electrolytes, limiting oxygen index (LOI) values were measured on JF-3 Oxygen index instrument with sheet dimensions of 100 mm × 10 mm × 3 mm following GB/T 2406.2-2009. Five samples were tested for each group. Vertical burning tests were measured according to GB/T 2408-2008 standard and the sample dimensions were 125 mm × 12.5 mm × 3.2 mm. The pore distribution was measured by Mercury intrusion porosimetry (MIP) (MicroActive AutoPore V 9600). Tensile and flexural tests of bi-continuous electrolytes at different temperatures were performed on the Instron 5567 machine following GB/T 2567-2008. The flexural tests of the PL_50_@SBICs performed on the Instron 5567 machine following Chinese Standard GB/T 1449-2005, and the dimensions of the PL_50_@SBICs samples used in the flexural (three-point bending) test were 30 mm × 15 mm × 1 mm, and the test was conducted using supports with a 16 mm span. The loading speed for the test was set at 2.0 mm/min.

The flexural modulus is calculated using the following equation:

$E_{f}$=$\frac{l^{3}\Delta P}{4{bh}^{3}\Delta S}$ (1)

Where $E_{f}$ represents flexural secant modulus of elasticity, l symbolizes the support span, b represents width of beam, h is the thickness of beam, ΔP is the force increment on the initial straight section of the force -deflection curve and ΔS is the deflection increment at the midpoint of support span corresponding to force increment ΔP.

The flexural stress is calculated using the equation:

σ =$\frac{3Pl}{2bh^{2}}$ (2)

where σ is the stress at the outer surface at mid-span, P is the applied force, l, b and h are the same as equation (1).

*Electrochemical Characterizations:* The Electrochemical Impedance Spectroscopy (EIS) was measured for the ionic conductivity using the electrochemical workstation (CHI660D, USA) in the temperature range of 25°C-200°C. Bi-continuous electrolytes were placed into an impedance test mold and clamped on both sides with stainless steel post electrodes. The testing frequency range was 10^-1^ to 10^6^ Hz. The ionic conductivity was calculated using the following equation:

σ=L/ (R_s_ 🞌 S). (3)

In the formula, σ represented the ionic conductivity, S symbolized the cross-section area of the sample, L represented the thickness of the employed electrolyte, and R_s_ was the measured impedance result.

The lithium-ion transference number ($t_{\mathrm{Li}^{+}}$) of the electrolyte was measured using the Li |PL_50_| Li cell and Li |ILE| Li cell by a combination of chronoamperometry and EIS methods. The $t_{\mathrm{Li}^{+}}$ was calculated as following equation:

$t_{\mathrm{Li}^{+}}$ = $\frac{I_{s}(\Delta V-I_{o}R_{o})}{I_{o}(\Delta V-I_{s}R_{s})}$ (4)

where $t_{\mathrm{Li}^{+}}$ represents the lithium-ion transport number of electrolytes; I_0_ and I_s_ are the initial and the steady state current measured by the chronoamperometry method; R_0_ and R_s_ are the initial and steady-state interfacial resistances obtained by the EIS test, respectively; ΔV is the applied polarization potential (10 mV).

The electrochemcial stability of PL_50_ was examined by measuring the linear sweep voltammetry (LSV) of a Li |PL_50_| SS cell and Li |ILE| SS cell at a scanning rate of 1 mV s^-1^. Cyclic voltammogram (CV) curves of PL_50_@SBICs were obtained on the electrochemical workstation (CHI660D, USA) at a scan rate of 5 mV s^−1^, and the current density was calculated based on the mass of LiFePO_4_ material. The galvanostatic charge/discharge tests were performed on the LAND CT3002A battery test system. The specific capacities of PL_50_@SBICs was calculated based on the mass of LiFePO_4_ material. All high-temperature measurements were carried out in an oven.


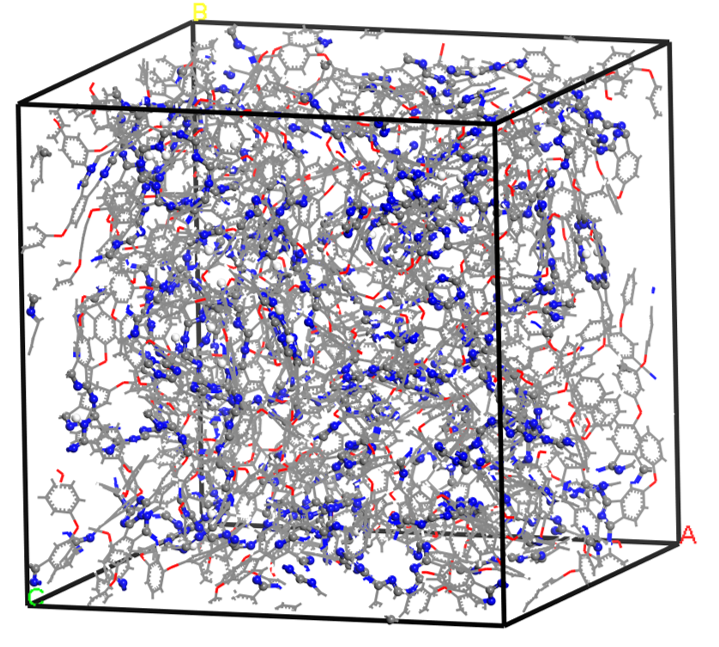


**Figure S1.** Crosslinking structure model of the cured PN resin.


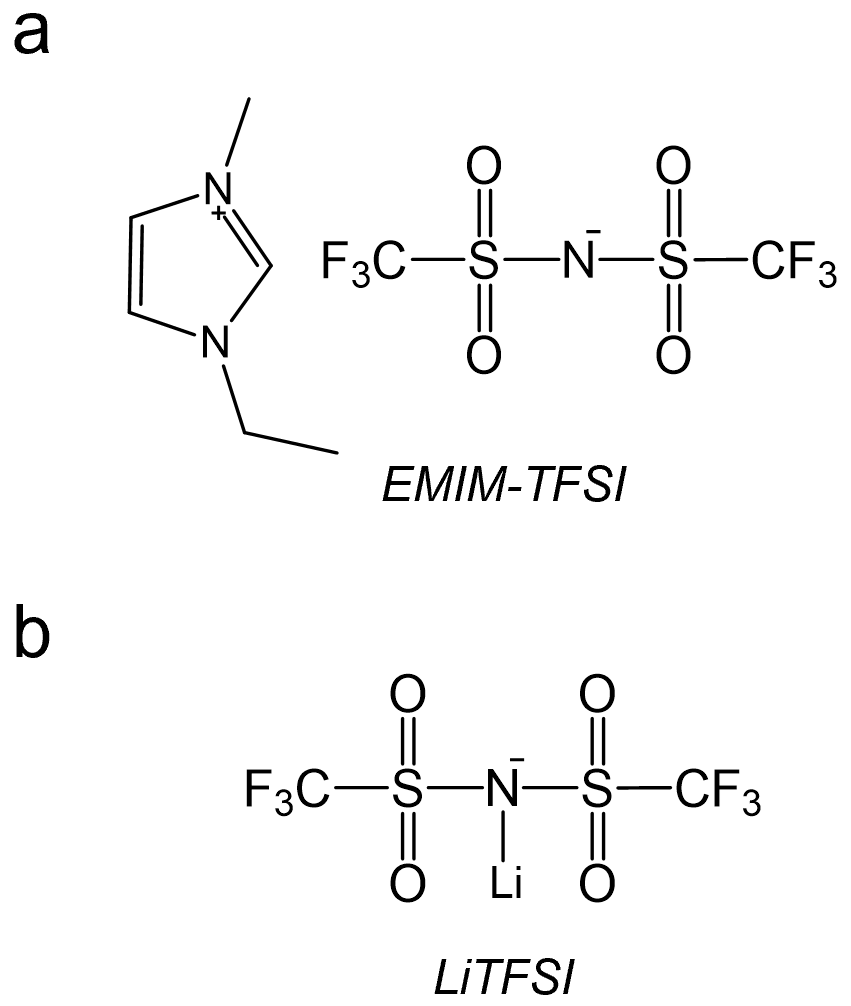


**Figure S2.** Chemical structure of the ionic liquid electrolyte.


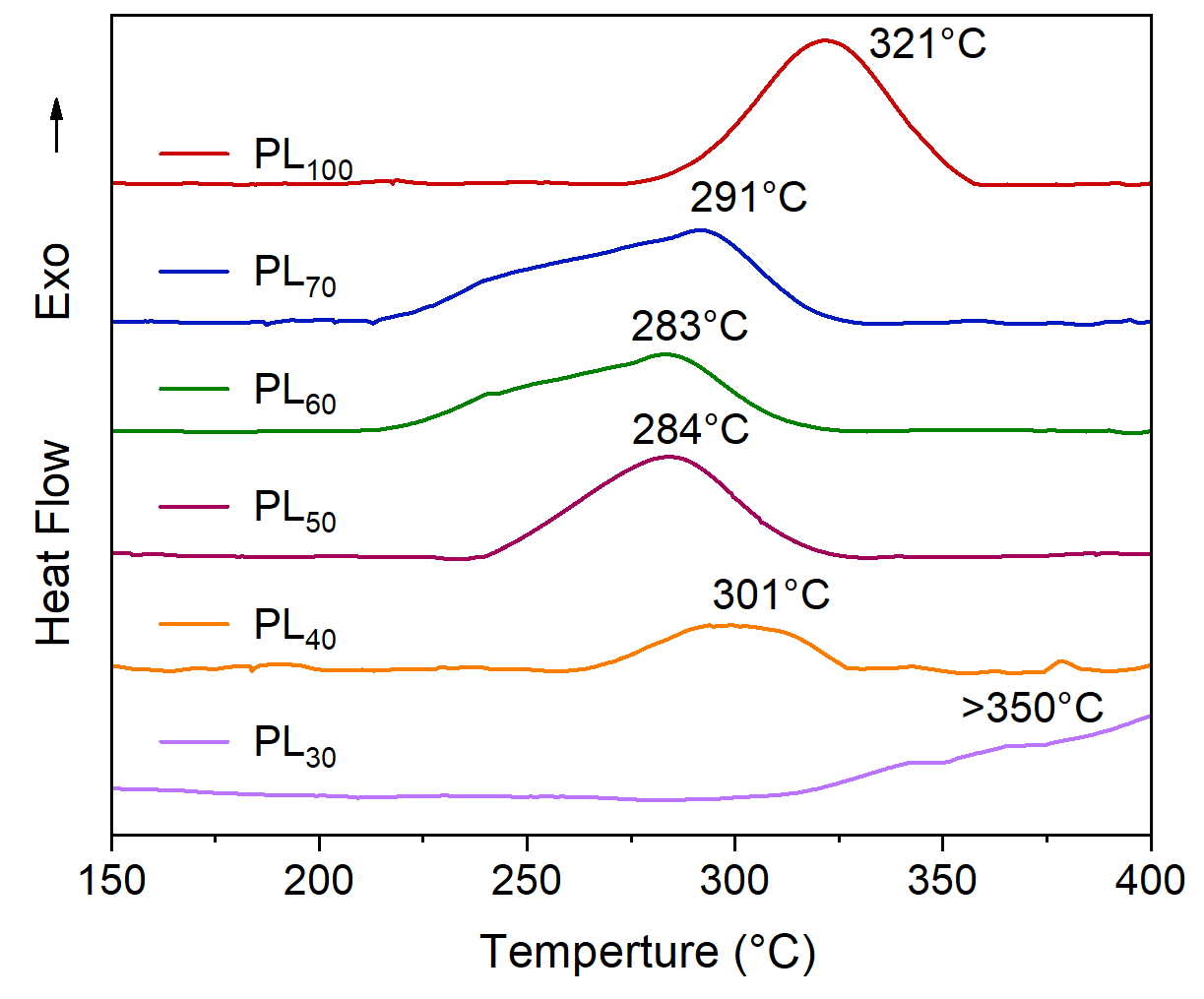


**Figure S3.** DSC measurement of precursors of PL_100_, PL_70_, PL_60_, PL_50_, PL_40,_ and PL_30_.

The maximum exothermic peak temperature of pure resin is 321°C. After adding ionic liquid electrolyte, the exothermic peaks temperature of bi-continuous electrolytes moves to lower temperature, and the maximum exothermic peak temperature of PL_70_, PL_60_, and PL_50_ is 291°C, 283°C, and 284°C. The results indicated that ionic liquid had catalytic effect on the curing reaction of phthalonitrile resin^[2]^. According to collision theory, the rate of chemical reactions depends on the frequency of collisions and the proportion of effective collisions between reactant molecules. An appropriate amount of ionic liquid electrolyte can catalyze the resin curing process, as seen in PL_70_ to PL_50_. However, in PL_40_, an excessive amount of ionic liquid electrolyte dilutes the monomer concentration, reducing the frequency of effective molecular collisions, which in turn slows down the curing reaction rate and raises the optimal curing temperature. Despite this, the exothermic peak temperature of PL_40_ resin remains lower than that of pure resin PL_100_, due to the catalytic effect of the ionic liquid electrolyte. Additionally, we included DSC measurements for the PL_30_ sample, which revealed that its exothermic peak temperature increased to over 350°C. This further confirms that an excessive amount of ionic liquid electrolyte negatively impacts the curing reaction rate of the resin monomer.


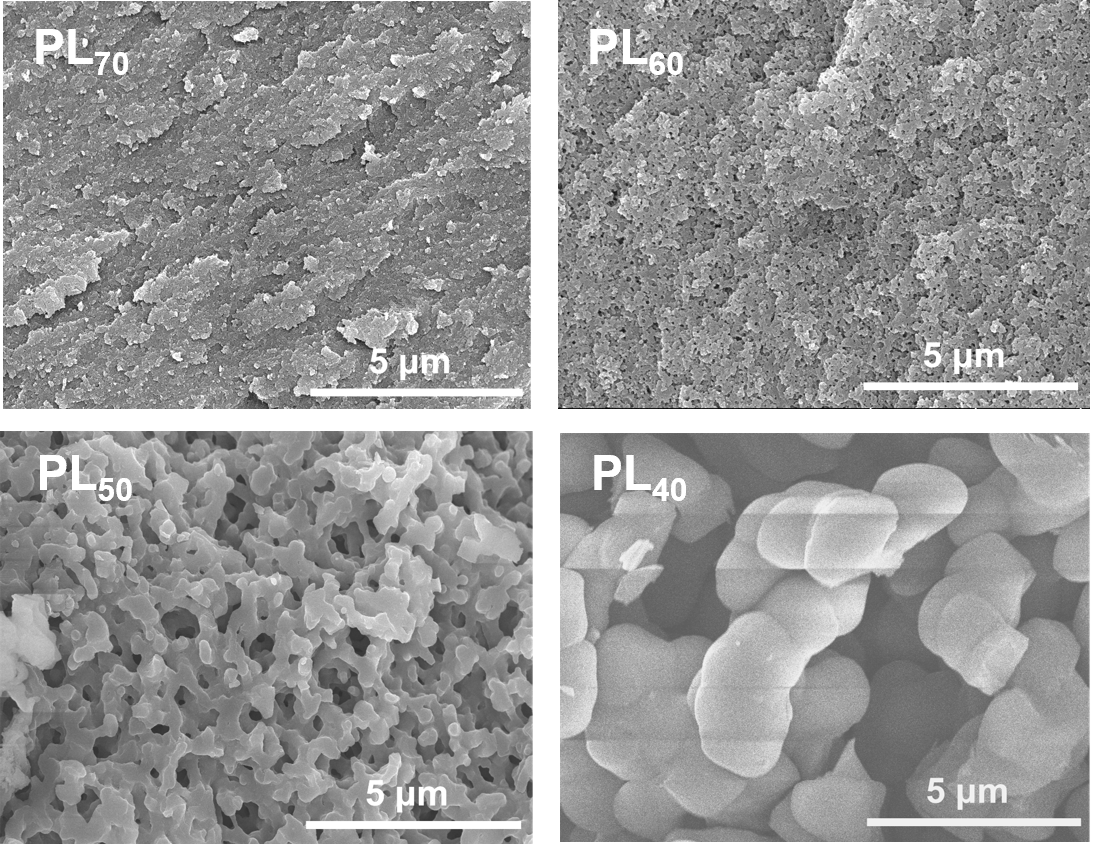


**Figure S4.** SEM images of PL_70_, PL_60_, PL_50_ and PL_40_.


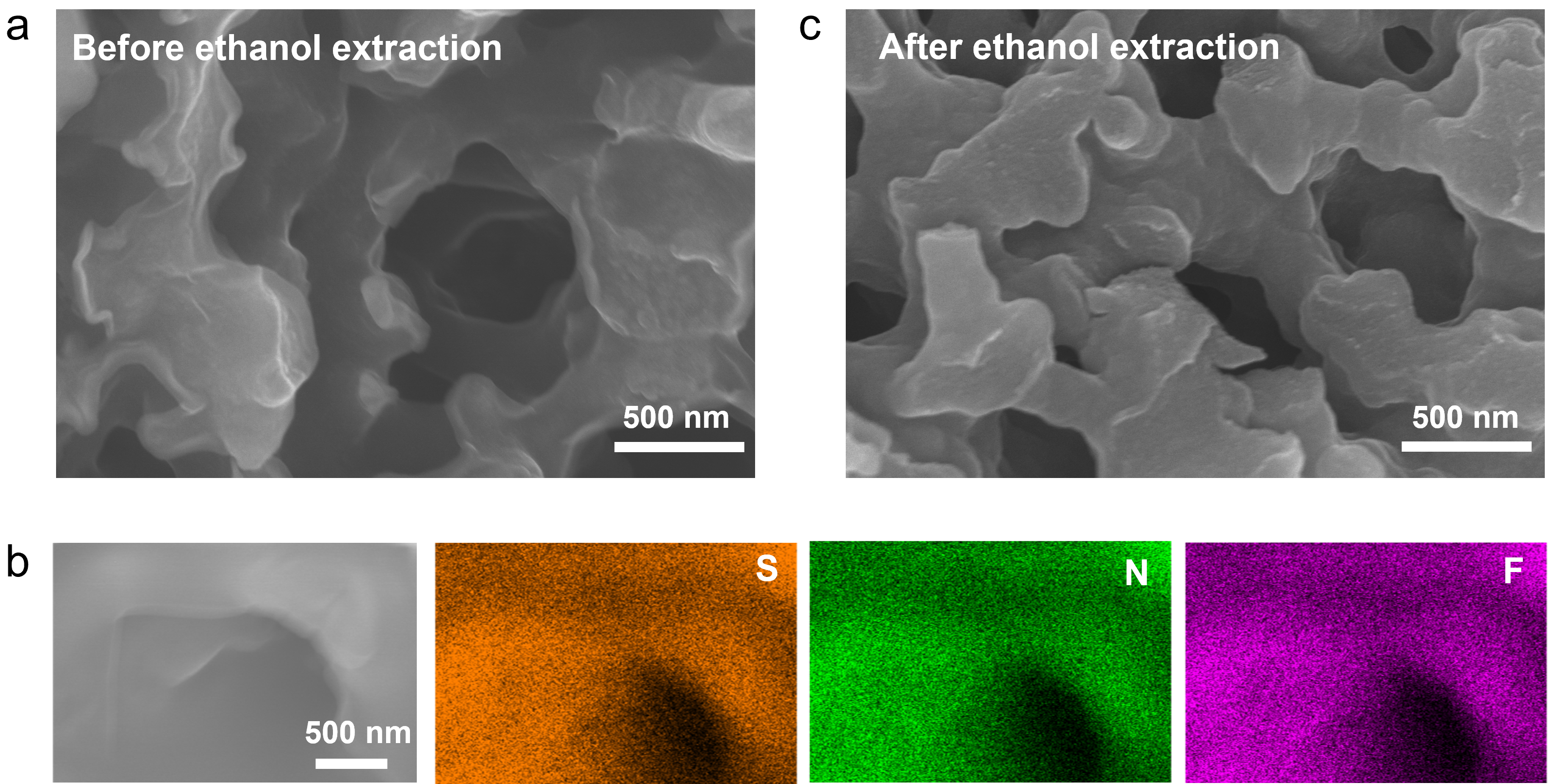


**Figure S5.** (a) SEM image and (b) energy-dispersive X-ray spectroscopy (EDS) mappings of PL_50_ before ethanol extraction. (c) SEM image of PL_50_ after ethanol extraction.

As shown in Figure S5a,b, SEM image and EDS mappings of the untreated sample clearly reveal the distribution of S, N, and F elements, which originate from the ILE, within the pores. After removing the ILE using ethanol extraction, the resulting sample revealed a clearer resin skeleton (Figure S5c). These results confirm that the skeleton in the bi-continuous structure is composed of PN resin, while the pores are filled with ILE.


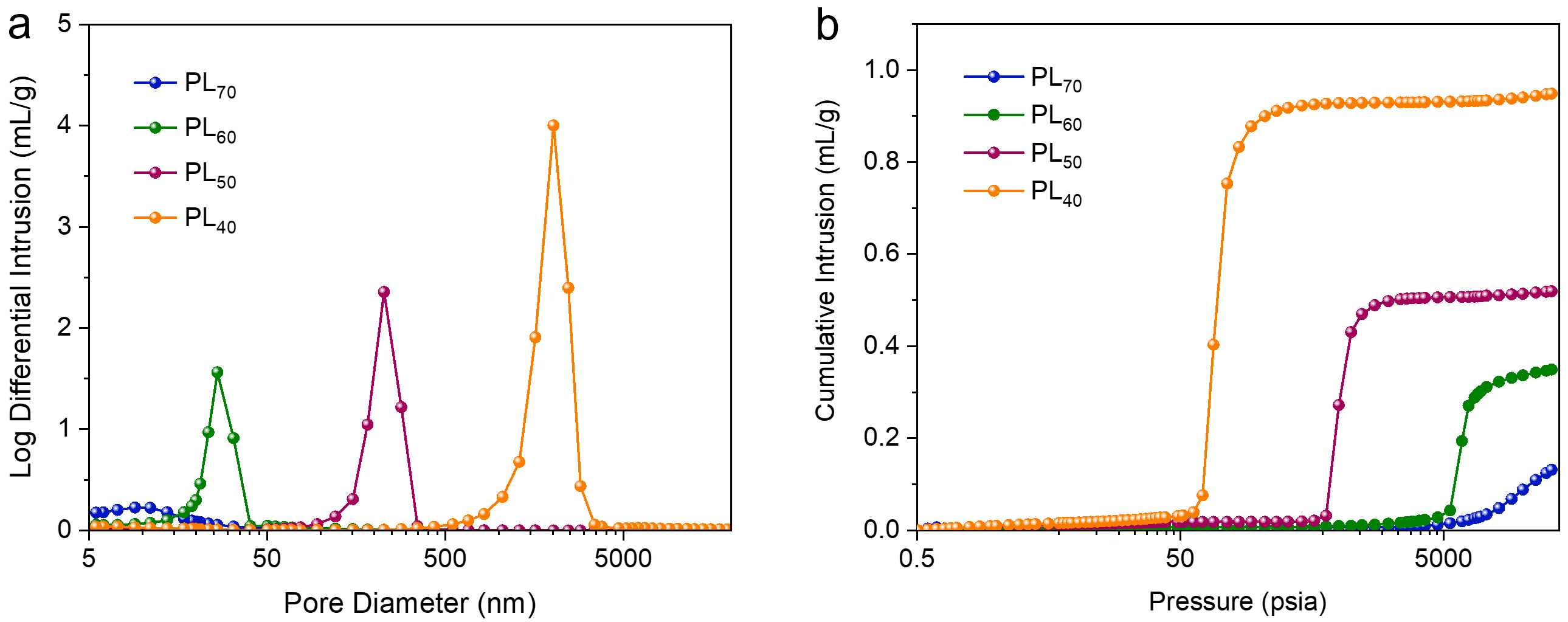


**Figure S6.** (a) Pore size distribution and (b) cumulative pore volume as a function of pressure from mercury intrusion porosimetry of PL_70_, PL_60_, PL_50_ and PL_40_. In order to more accurately measure the pore size distribution of the bi-continuous electrolytes, the samples tested by mercury intrusion porosimetry were multiple discs with 10 mm diameter and 2 mm height.


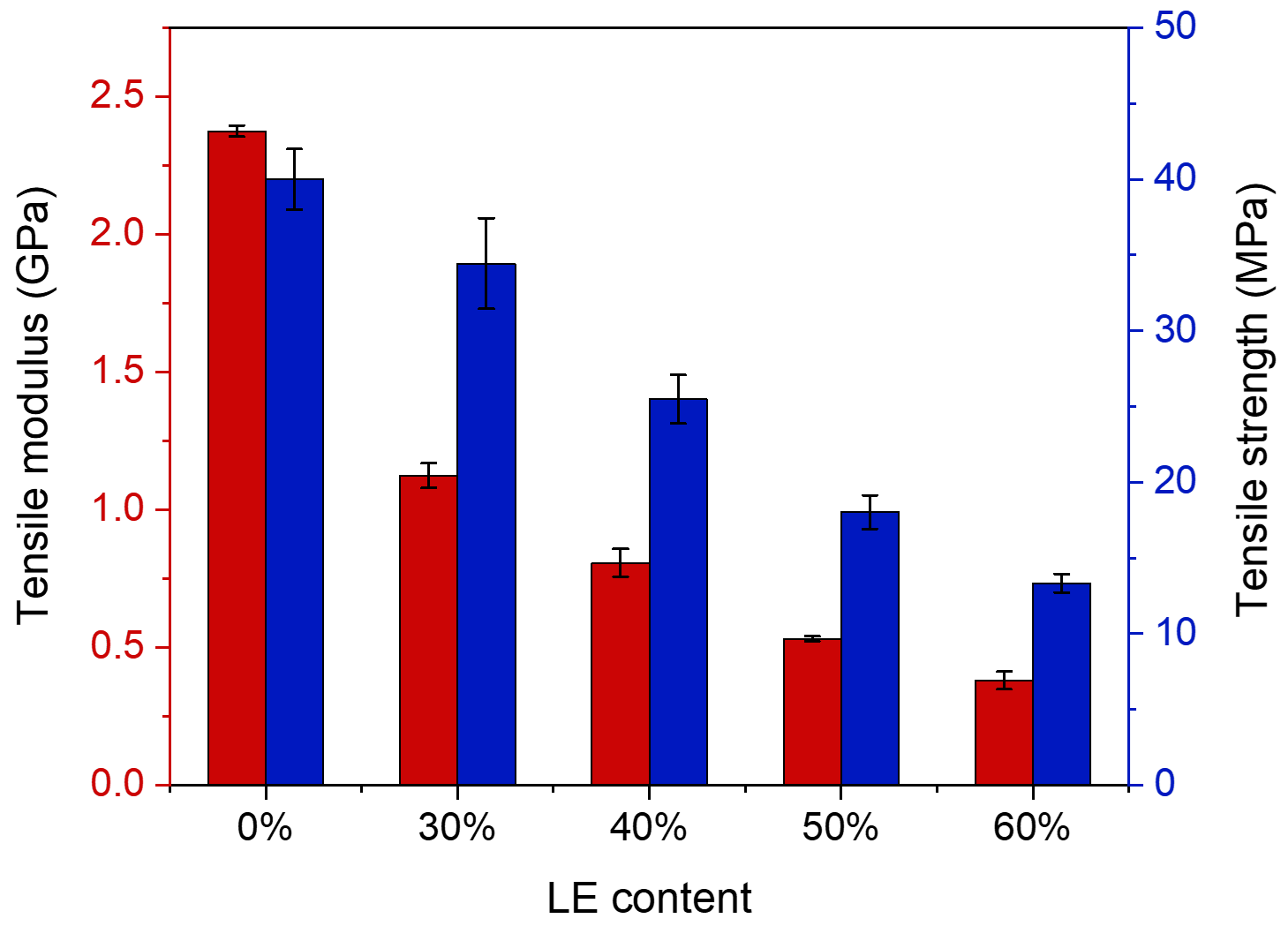


**Figure S7.** Tensile modulus and strength of different bi-continuous electrolytes.


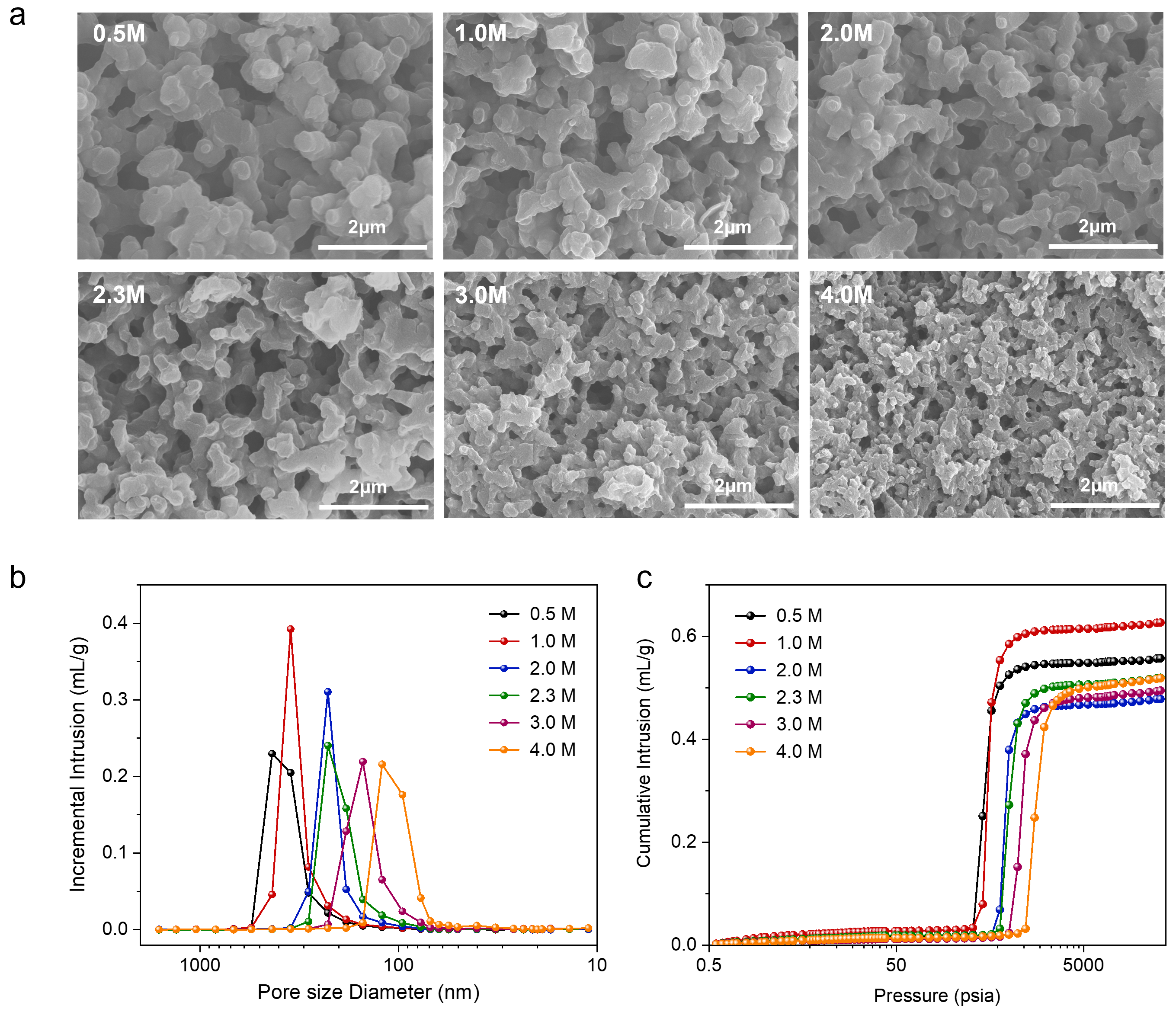


**Figure S8.** (a) SEM images of PL_50_ containing different LiTFSI concentrations. (b) Pore size distribution and (c) cumulative pore volume as a function of pressure from mercury intrusion porosimetry of PL_50_ containing different LiTFSI concentrations. The high polarity nitrile group of PN resin can increase miscibility of the resin with ionic liquid electrolyte, which greatly reduces the solubilizing effect of lithium salts so that the bi-continuous phase structure can be formed at low lithium salt concentration^[3]^. The increase in the viscosity of the ionic liquid electrolyte decreases the fluidity of the liquid phase, which kinetically decreases the rate of phase separation during curing. As a result, the bi-continuous phase size increases with lithium salt concentration.


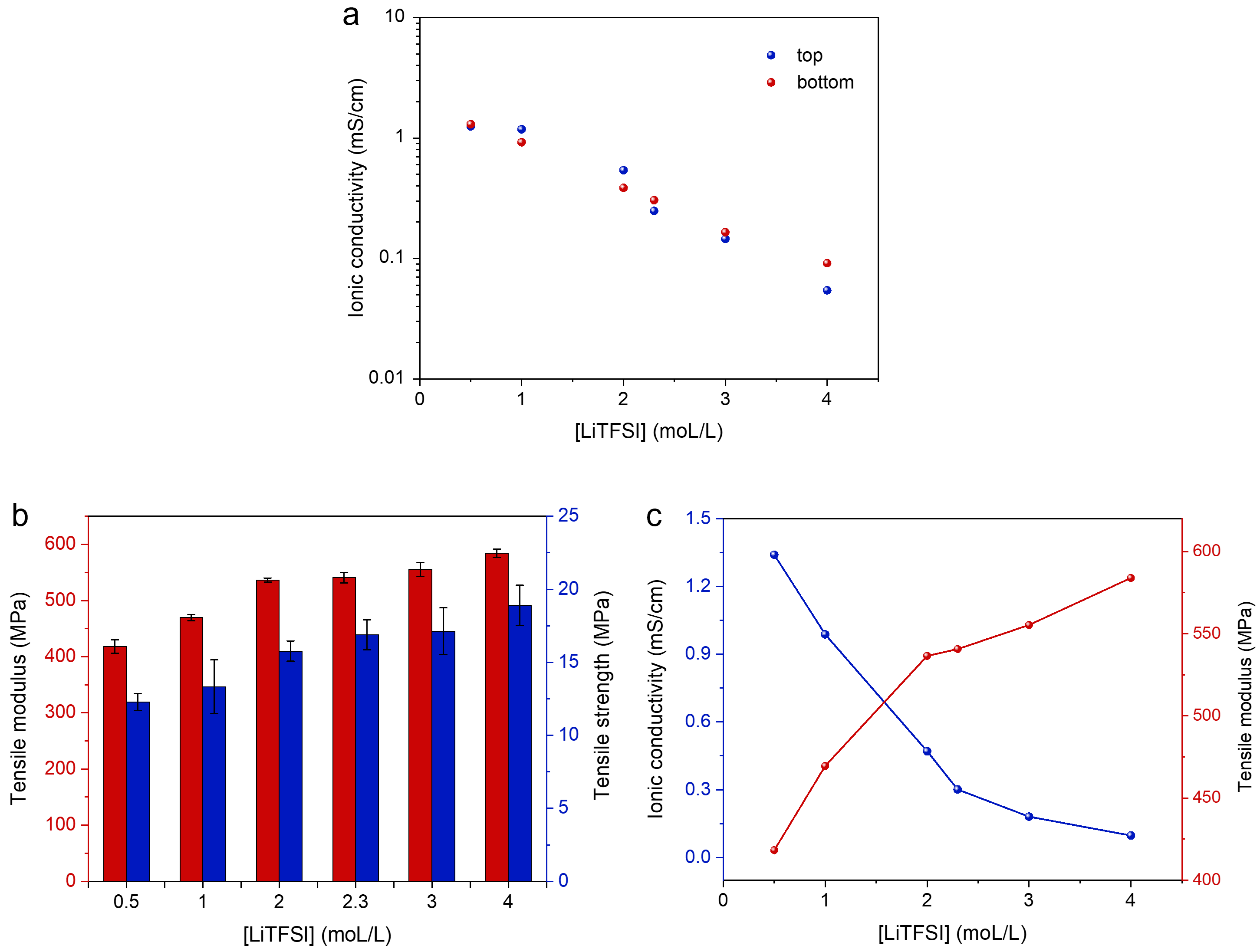


**Figure S9.** (a) Ionic conductivity at top and bottom of cylinder PL_50_ sample containing different LiTFSI concentrations. (b) Effect of LiTFSI concentration on Tensile modulus and strength for PL_50_. (c) Ionic conductivity and Tensile modulus as functions of LiTFSI concentration in the PL_50_. Samples were cured in the vertical position using cylindrical moulds with diameter 11 mm and height 50 mm. The ionic conductivity of the top and bottom discs of the cylinder samples containing different concentrations of lithium salts is basically similar, which indicates the homogeneity of the overall structure of the sample. The bi-continuous phase size increases with lithium salt concentration, which leads to an increase in mechanical strength and a decrease in ionic conductivity.


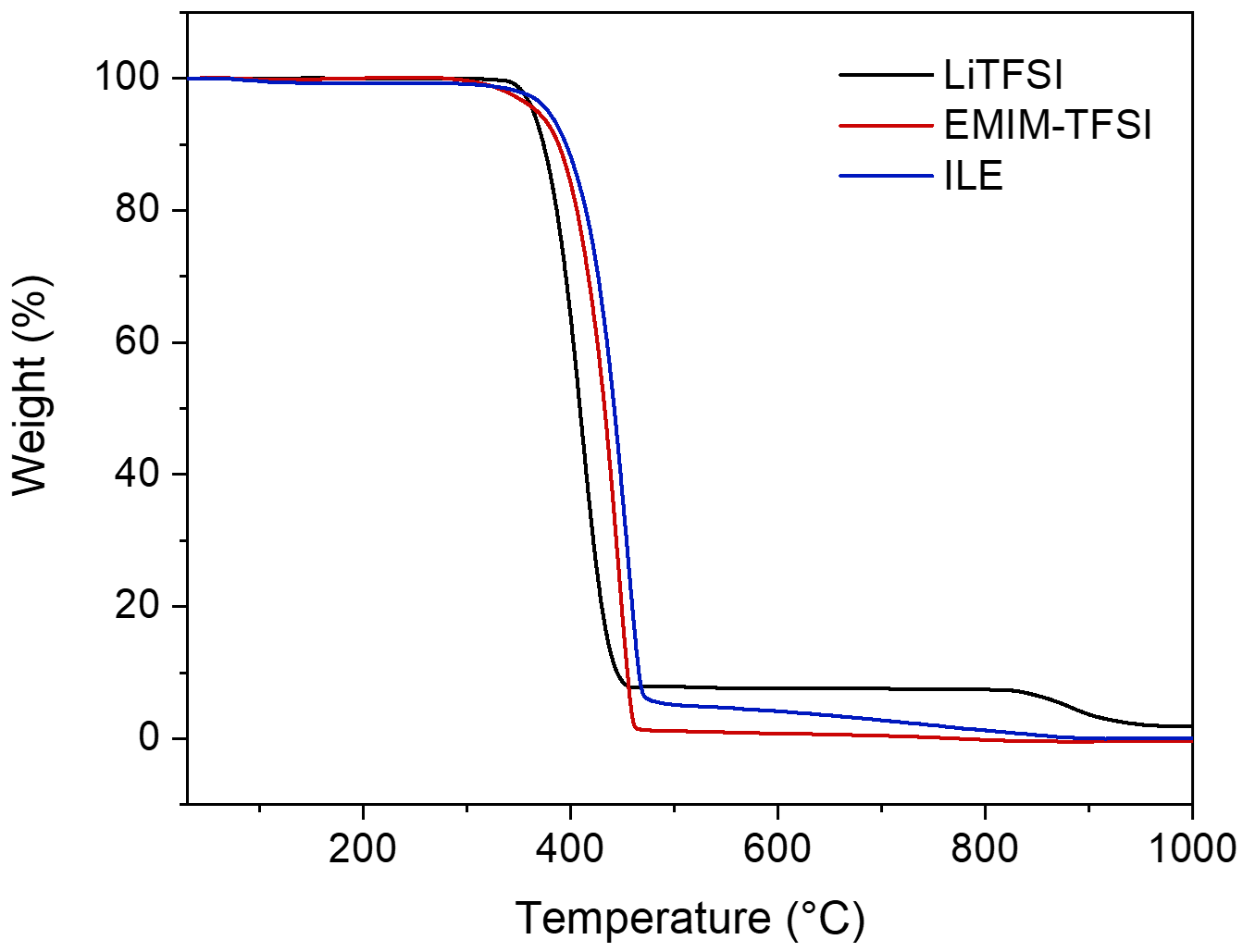


**Figure S10.** TGA curves for LiTFSI, EMIM-TFSI and the ILE.


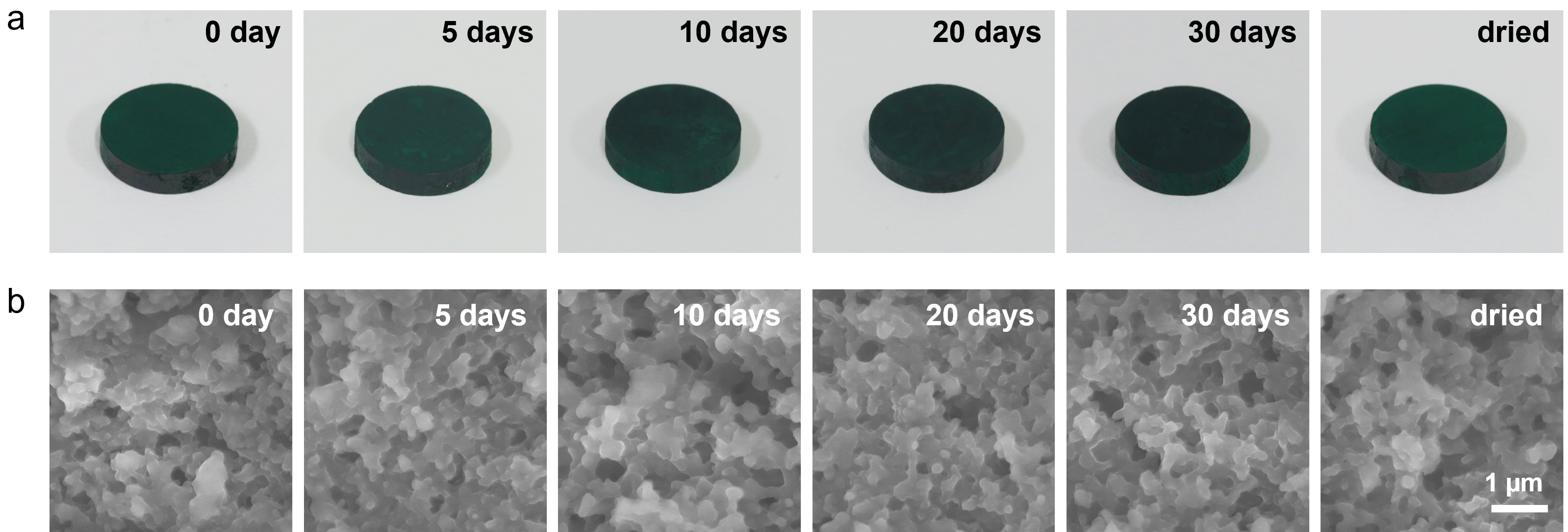


**Figure S11.** Photographs (a) and SEM images (b) of the PL_50_ electrolyte after being placed in the air atmosphere for different periods.

We examined the structural evolution of the bi-continuous electrolyte during exposure to the atmosphere using optical and SEM images. As shown in Figure S11a, the electrolyte surface darkened over time due to the water absorption by Li-TFSI after being exposed to the air. However, no liquid electrolyte leakage was observed in the PL_50_ electrolyte within 30 days. This phenomenon can be attributed to the nitrile groups or nitrogen atoms in the phthalocyanine and triazine structures of the PN resin, which can adsorb ionic liquids, lithium salts, and other cations through Lewis acid-base interactions. Additionally, the nanoscale pores in the PN resin skeleton increased the specific surface area, enhancing the adsorption of liquid electrolytes. The electrolyte color returned to normal after high temperature drying. As shown in Figure S11b, the SEM images indicate that no collapse or other changes occurred in the PL_50_ electrolyte within 30 days.


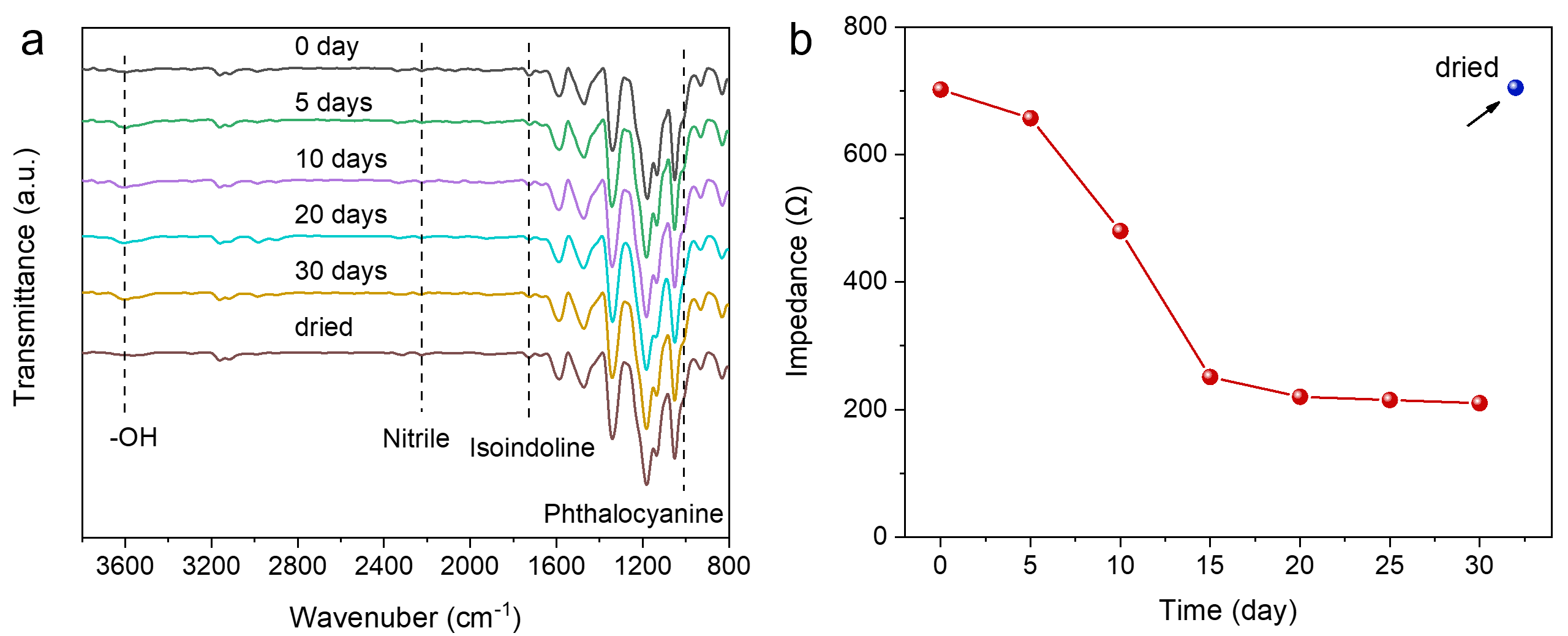


**Figure S12.** FTIR spectra (a) and the impedance spectra (b) of the PL_50_ electrolyte after being placed in the air atmosphere for different periods.

As shown in Figure S12a, no new peaks appeared in the FTIR spectra, confirming the chemical stability of the PL_50_ electrolyte in an air atmosphere. Figure S12b shows the change of impedance value of the PL_50_ over different periods of exposure to the air. During the first 0-15 days, the impedance gradually decreases, which may be attributed to the water absorption by Li-TFSI, as the water has a lower viscosity and a higher polarity than the ionic liquid electrolyte, which is conducive to the dissociation of lithium salts. However, due to the coordination between cations and the PN resin, excessive water absorption is prevented, and the impedance eventually stabilizes. After drying, the impedance returns to its initial value (0 days), confirming the stability of the PL_50_ electrolyte against water and air.


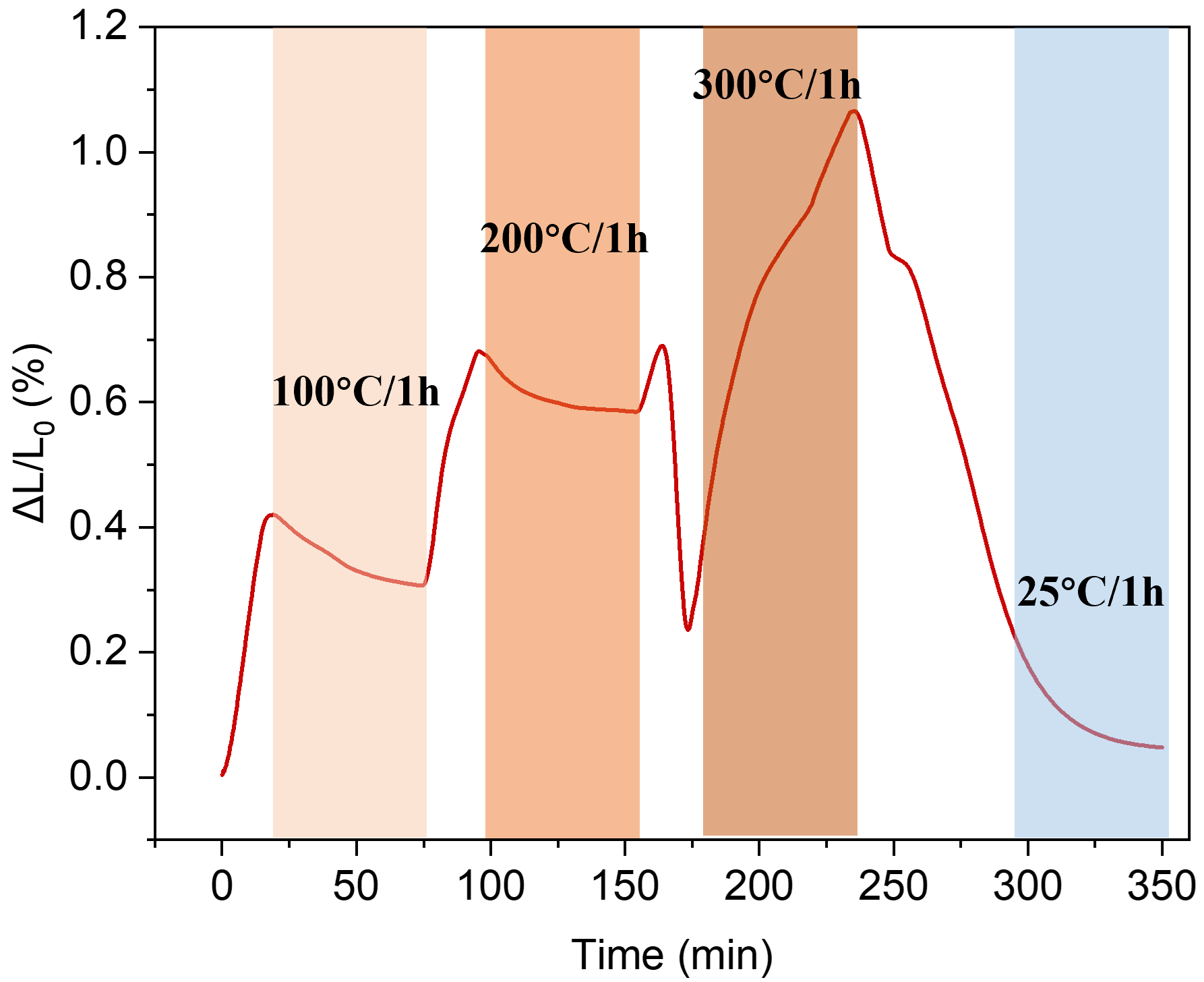


**Figure S13.** Thermal expansion behavior of PL_50_ sample heated at 100℃, 200℃, and 300℃for 1 h and cooled to 25°C for 1 h in air.

The PL_50_ samples were heated from 30°C to 300°C at a rate of 5°C/min in an air atmosphere, with isothermal holds at 100°C, 200°C, and 300°C for 1 h, respectively, then cooled to 25°C at a rate of 5°C/min and isothermal holds at 25°C for 1 h. As shown in Figure S13, the thermal expansion rate of the sample is only about 0.31% after isothermal at 100°C for 1 h. The thermal expansion rate increases to 0.58% after 1 h at 200°C. As the temperature rises further, the thermal expansion rate initially drops due to the softening of the sample, at which point the molecular chain segments in the sample begin to move, transitioning from a glassy state to a highly elastic state. The thermal expansion rate then increases rapidly, reaching 1% after 1 h of heating at 300°C, and the size change is very small at high temperature. After the sample was cooled to 25°C and isothermal hold for 1h, the thermal expansion rate dropped to 0.05%, indicating that the sample size almost recovered to the initial value. In summary, PL_50_ electrolyte has excellent structural stability and thermal stability.


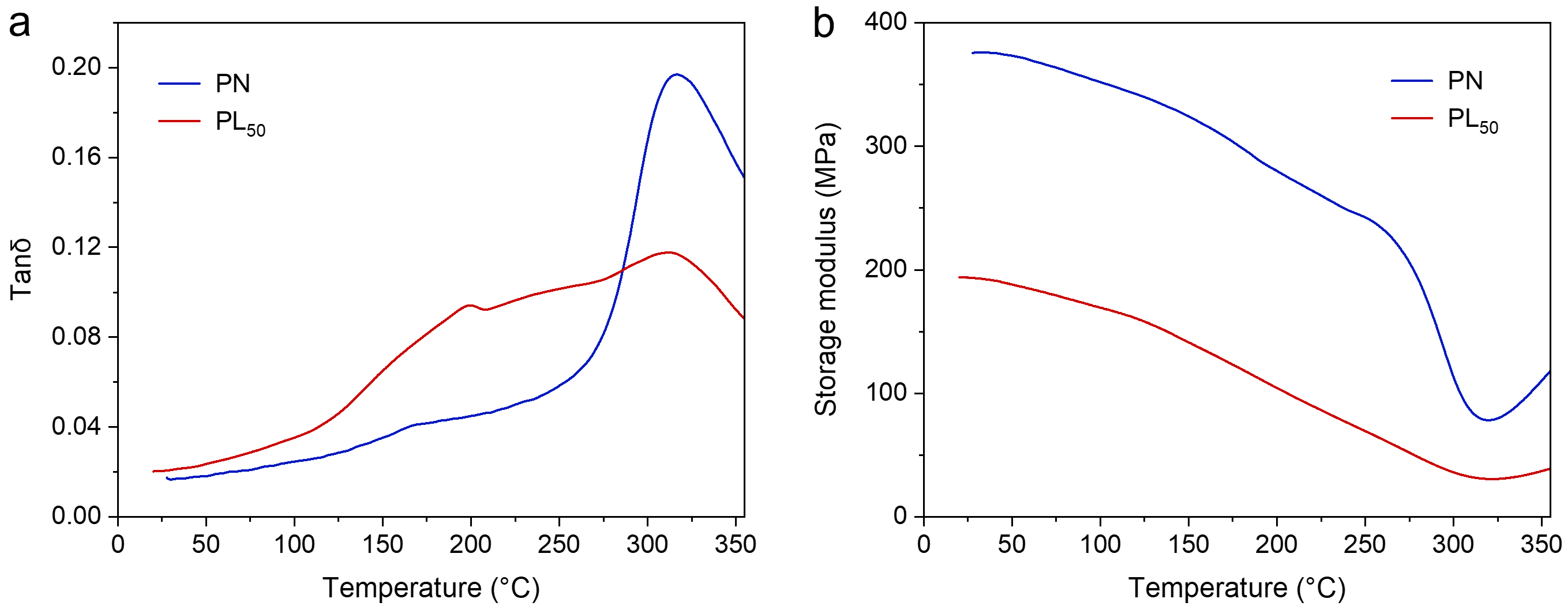


**Figure S14.** Dynamic mechanical analysis (DMA) of PN resin and PL_50_ containing 1.0 mol/L LiTFSI in ionic liquid. (a) The tan δ curves. (b) Storage modulus curves.


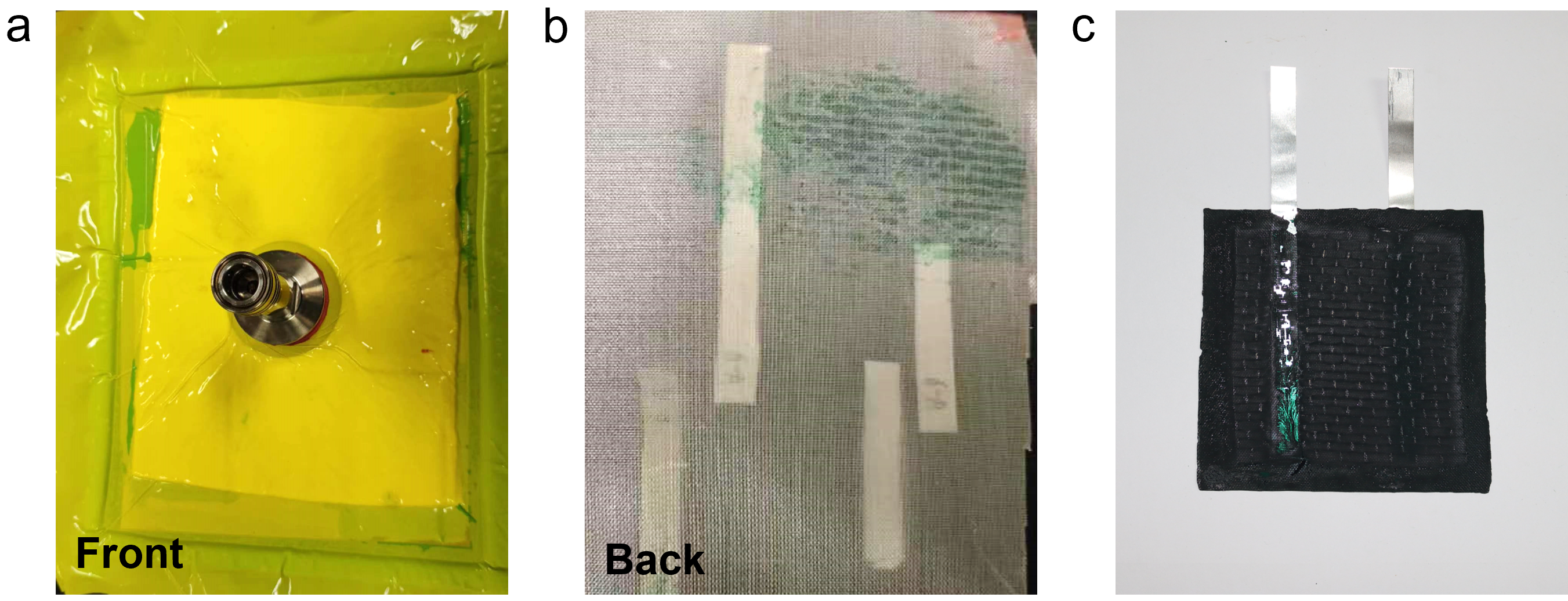


**Figure S15.** The vacuum bagging process of PL_50_@SBICs.


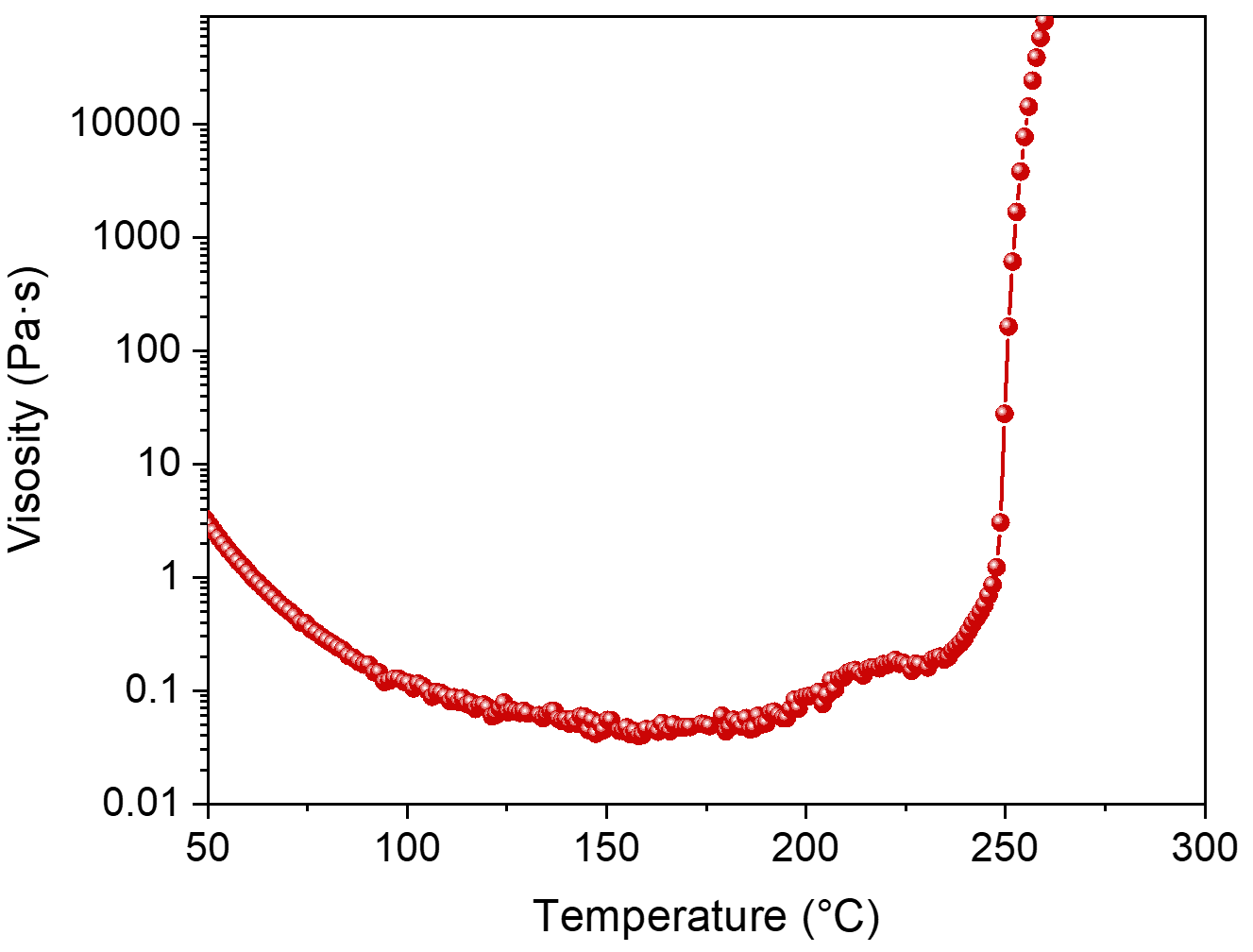


**Figure S16.** Viscosity of PL_50_ changed as function of temperature.


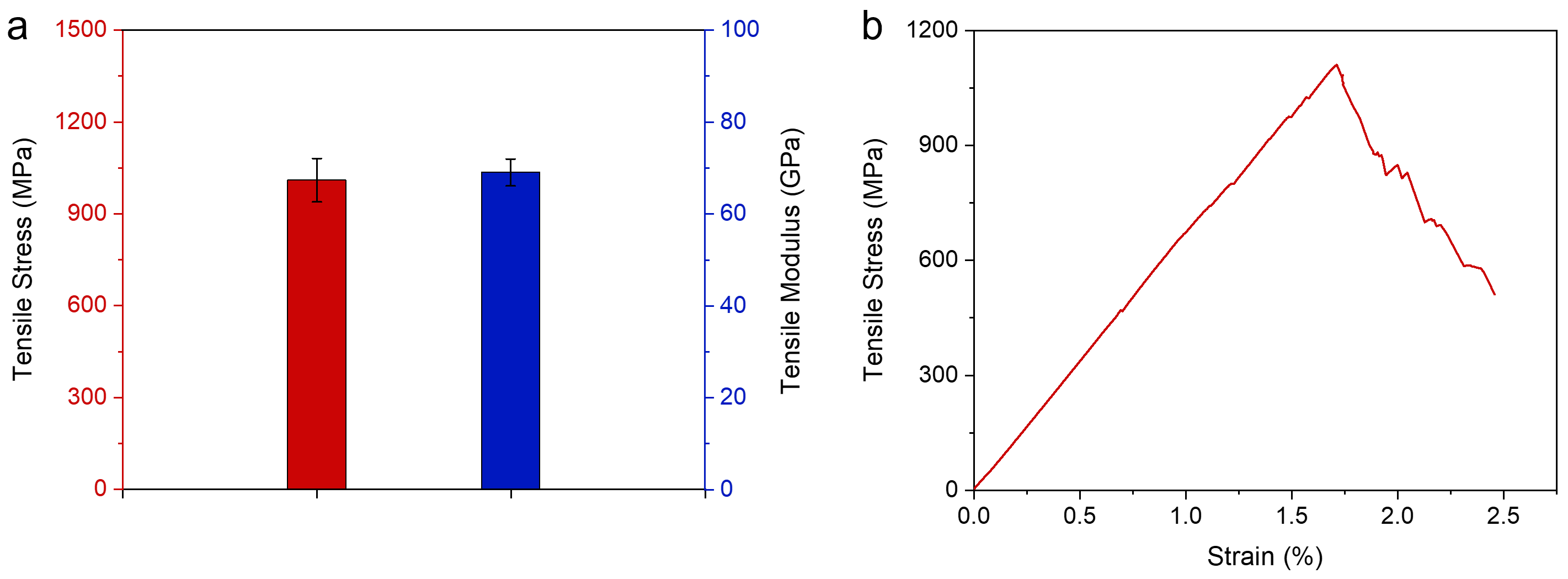


**Figure S17.** (a) Tensile modulus and strength of PL_50_@SBICs. (b) Tensile stress-strain curve of PL_50_@SBICs.

The tests were conducted using a testing machine (E45.305, MTS) with sample dimensions of 150 mm × 10 mm × 1 mm, and the loading testing was set at 2.0 mm/min. As shown in Figure S17a, the PL_50_@SBICs exhibited a tensile strength of 1010±71 MPa and a tensile modulus of 69.04±2.9 GPa at 25°C. The remarkable mechanical properties of the PL_50_@SBICs can be attributed to the synergistic effect of carbon fibers as reinforcement and PL_50_ as the polymer matrix. As shown in Figure S17b, the stress-strain curve exhibits linear elastic behavior, with almost no damage micro-cracks observed before reaching the maximum stress, indicating excellent adhesion between the PL_50_ electrolyte and the carbon fiber electrodes. The sharp drop in the curve corresponds to the rupture of the carbon fibers, which are the primary load-bearing component.


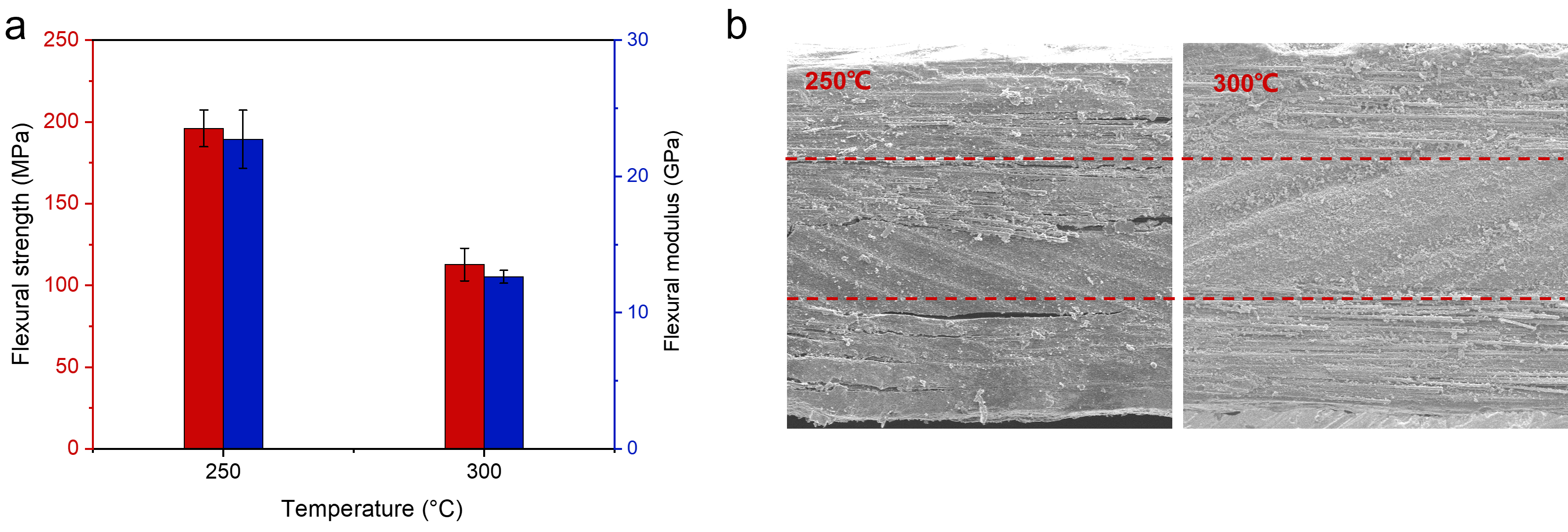


**Figure S18.** (a) Flexural modulus and strength of PL_50_@SBICs at 250°C and 300°C. (b) Cross-sectional SEM images of PL_50_@SBICs after flexural testing at 250°C and 300°C. As the test temperature reaches *T_g_* of PL_50_ at 300°C, the resin matrix softens and deforms, causing the spline to fail due to macroscopic deformation.


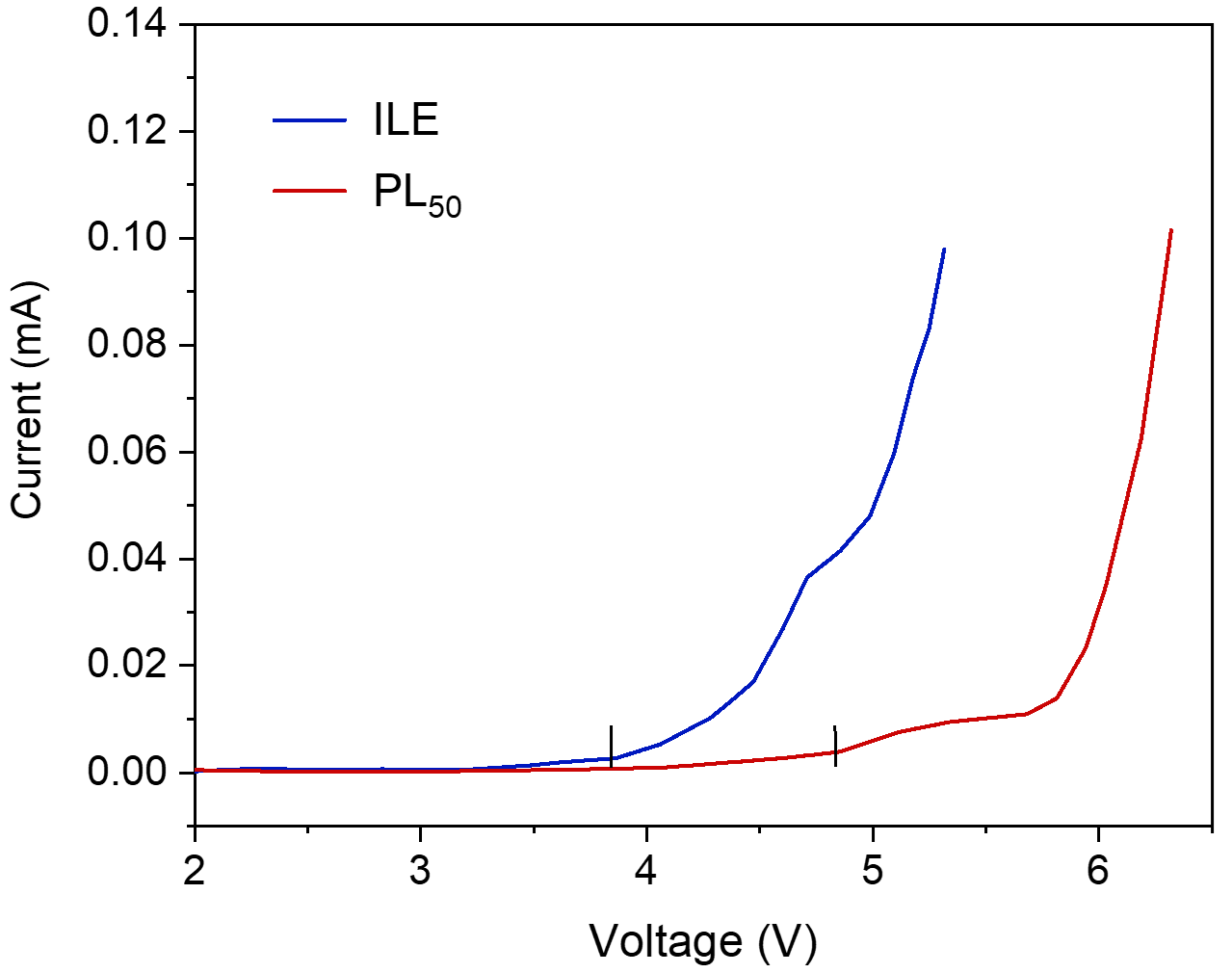


**Figure S19.** Electrochemical stability of the PL_50_ and ILE, obtained by linear sweep voltammetry (LSV).


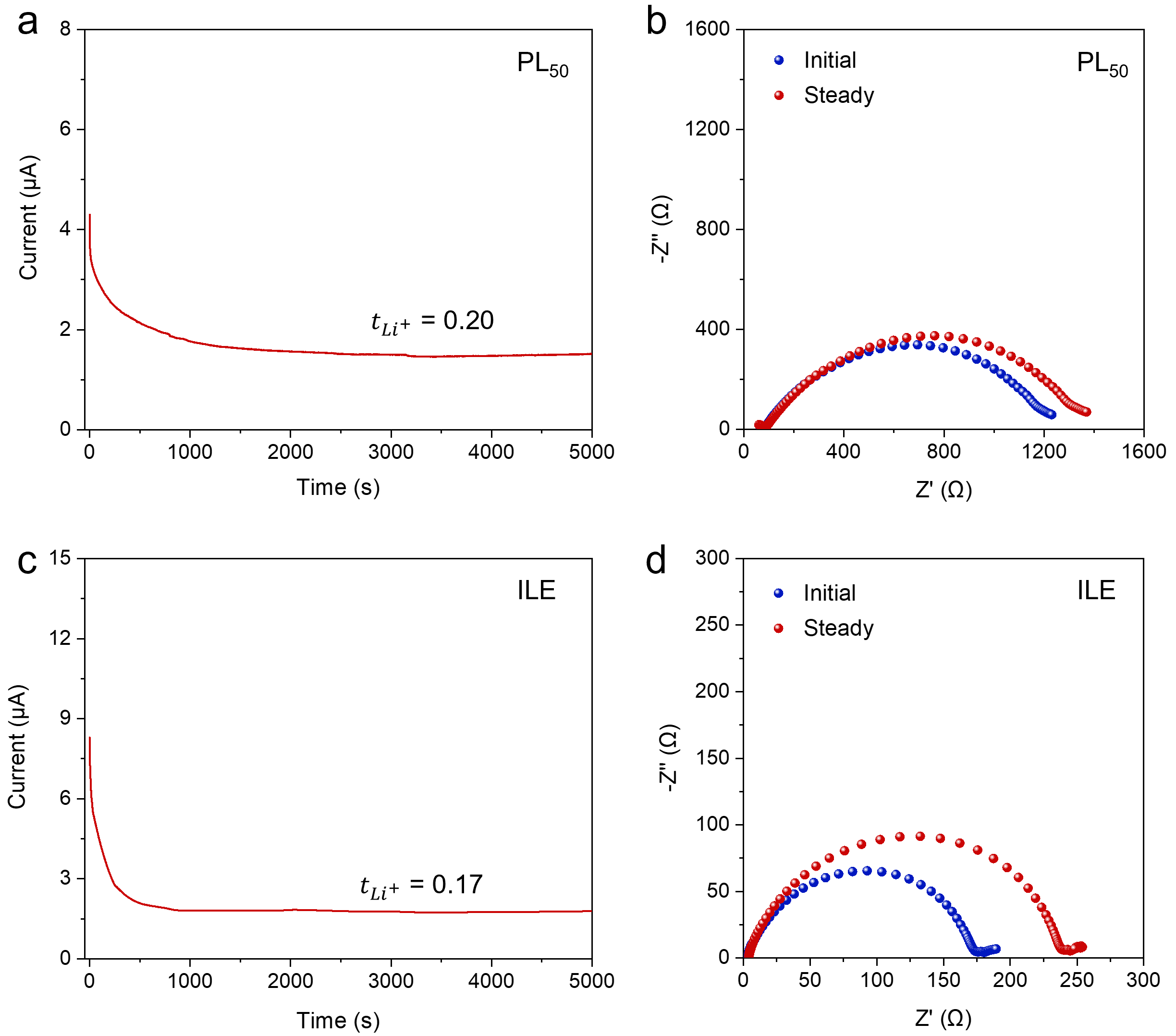


**Figure S20.** (a) Polarization curve and (b) initial and steady-state impedance spectra of PL_50_. (c) Polarization curve and (d) initial and steady-state impedance spectra of ILE.


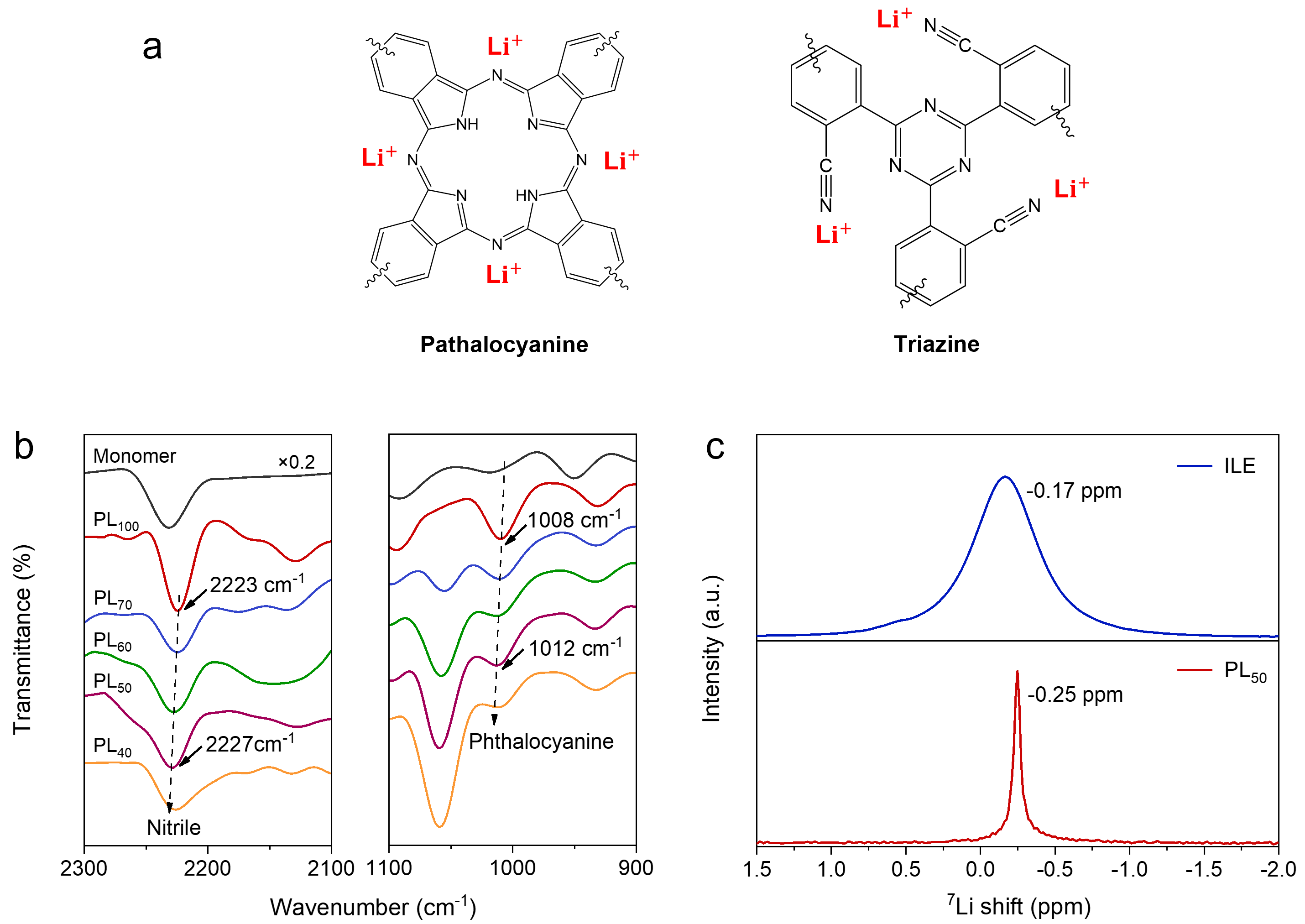


**Figure S21.** (a) Schematic illustration of the positive charged triazine and pathalocyanine. (b) FTIR spectra of the PL_50_ electrolyte. (c) ^7^Li solid state NMR spectra of ILE and PL_50_ electrolyte.

In PL_50_ electrolyte, the resin structure contains unreacted nitrile groups, nitrogen-containing triazine rings, and phthalocyanine structures (Figure S21a). N atoms can provide one extra pair of p-electron as necessary donor attracting Li ions by Lewis acid-base interaction, thereby promoting the dissociation of lithium salts, and improving the lithium transfer number ($t_{\mathrm{Li}^{+}}$). To further illustrate this, we have supplemented the Fourier transform infrared spectroscopy (FTIR) spectra and nuclear magnetic resonance (NMR) for both PL_50_ and ionic liquid electrolytes. As shown in Figure S21b, with the addition of ionic liquid electrolyte, the characteristic peak of phthalocyanine in the structural electrolyte migrates from 1008 cm^-1^ to 1012 cm^-1^, and the increase in wave number proves the coordination between N atoms and Li ions. The characteristic peak of the triazine ring structure is about 1363 cm^-1^, which coincides with the characteristic peak of the ionic liquid electrolyte and cannot be judged. However, the characteristic peak of the unreacted nitrile group due to the steric hindrance in the structural electrolyte increased from 2223 cm^-1^ to 2227 cm^-1^, indicating that the nitrile group and Li ion attract each other due to Lewis acid-base interaction. Analyzed by ^7^Li solid-state NMR, the signal in PL_50_ is shifted from -0.17 to -0.25 ppm compared with the liquid electrolyte (Figure S21c), which indicates the chemical circumstance of the lithium-ion has been changed by PN resin. In summary, the unreacted nitrile groups and the nitrogen atoms in the cross-linked structure attract lithium ions due to Lewis acid-base action, thus promoting the dissociation of lithium salts, making the lithium transfer number ($t_{\mathrm{Li}^{+}}$) of PL_50_ higher than the ionic liquid electrolyte.


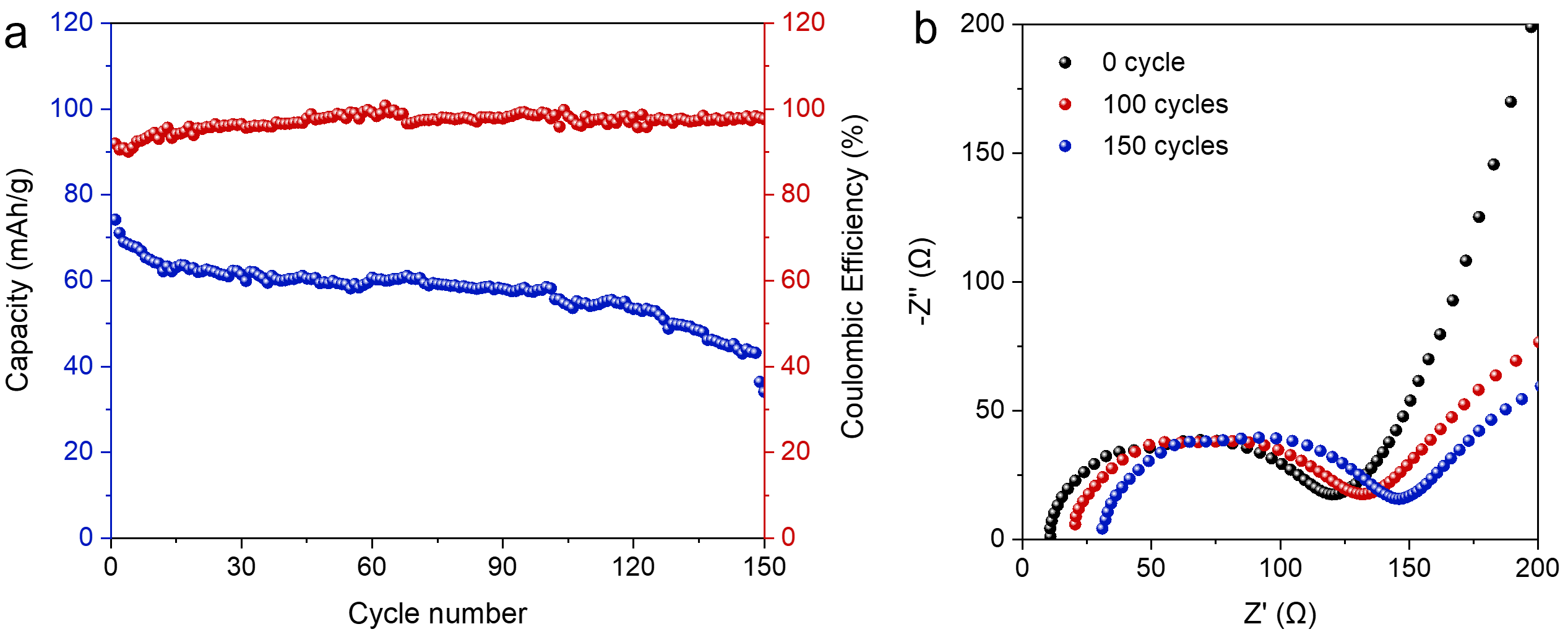


**Figure S22.** (a) Cycling performance of PL_50_@SBICs at a rate of 0.2 C, cycled at 25°C. (b) The EIS of PL_50_@SBICs before and after 100 cycles and 150 cycles.

Figure S22 indicate that while the Coulombic efficiency remained stable throughout the testing, the specific capacity began to gradually decline after the 120th cycle, eventually dropping to around 40 mAh/g by the 150th cycle. However, the impedance value increased only slightly from 134 Ω to 146 Ω over 120 to 150 cycles. Therefore, we speculate that this decline in specific capacity may be attributed to the poor cycling performance of the unmodified carbon fibers.


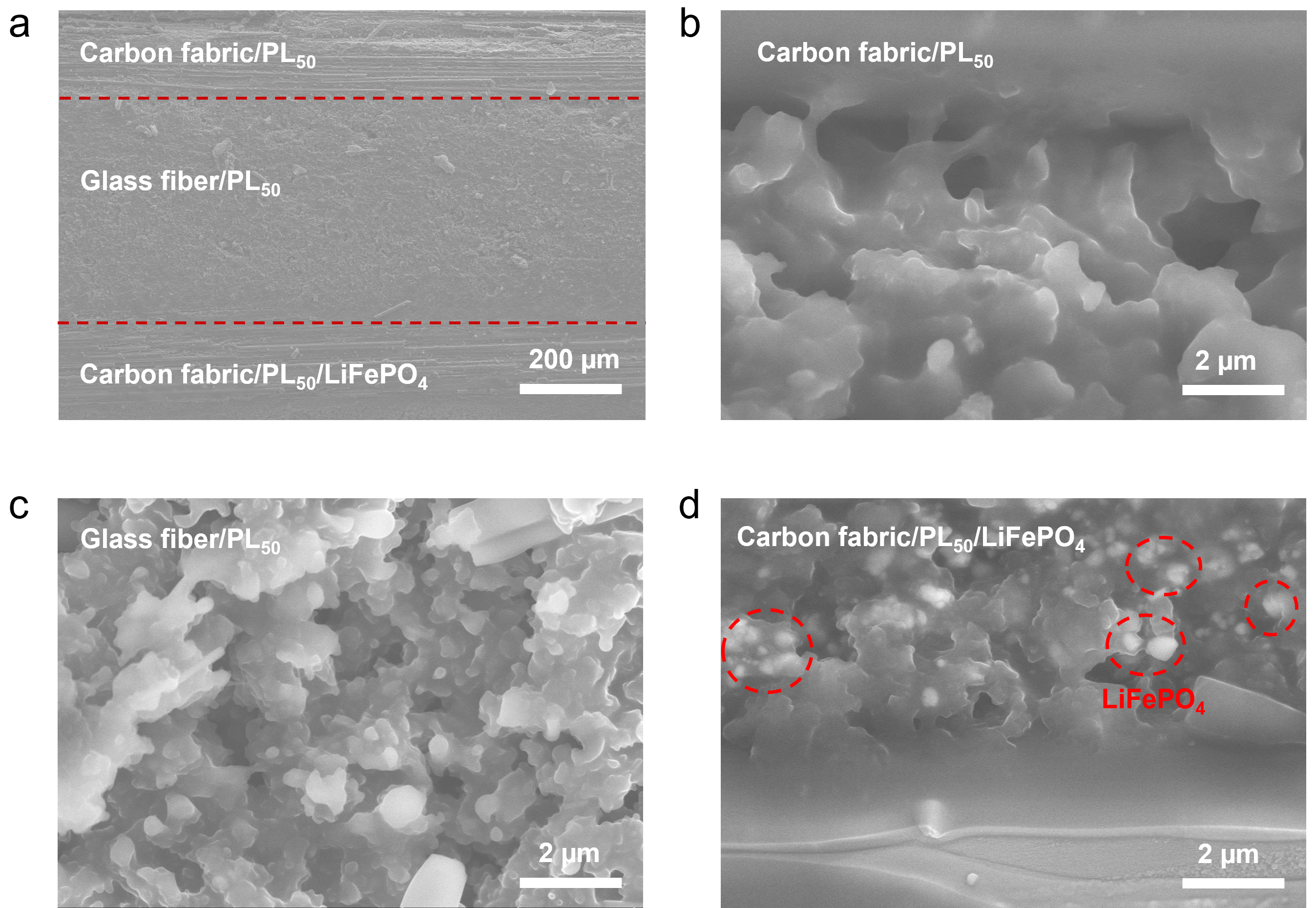


**Figure S23.** (a) Cross-sectional SEM image of PL_50_@SBICs after cycling. Scale bar is 200 µm. (b) SEM images of the interface between CF and PL_50_. (c) SEM image of PL_50_. (d) SEM images of the interface between LiFePO_4_ coated on CF and PL_50_.

On a macroscopic level, due to the excellent mechanical properties of the bi-continuous structure electrolyte, the SBICs did not exhibit any deformation, bulging, or leakage after cycling. On a microscopic level, SEM images of the battery cross-section reveal that the cycled battery maintained a uniform composite structure without obvious interlayer separation, with the cathode and anode electrodes tightly adhered to the PL_50_ electrolyte. Furthermore, the microstructure of the PL_50_ electrolyte remained largely unchanged.


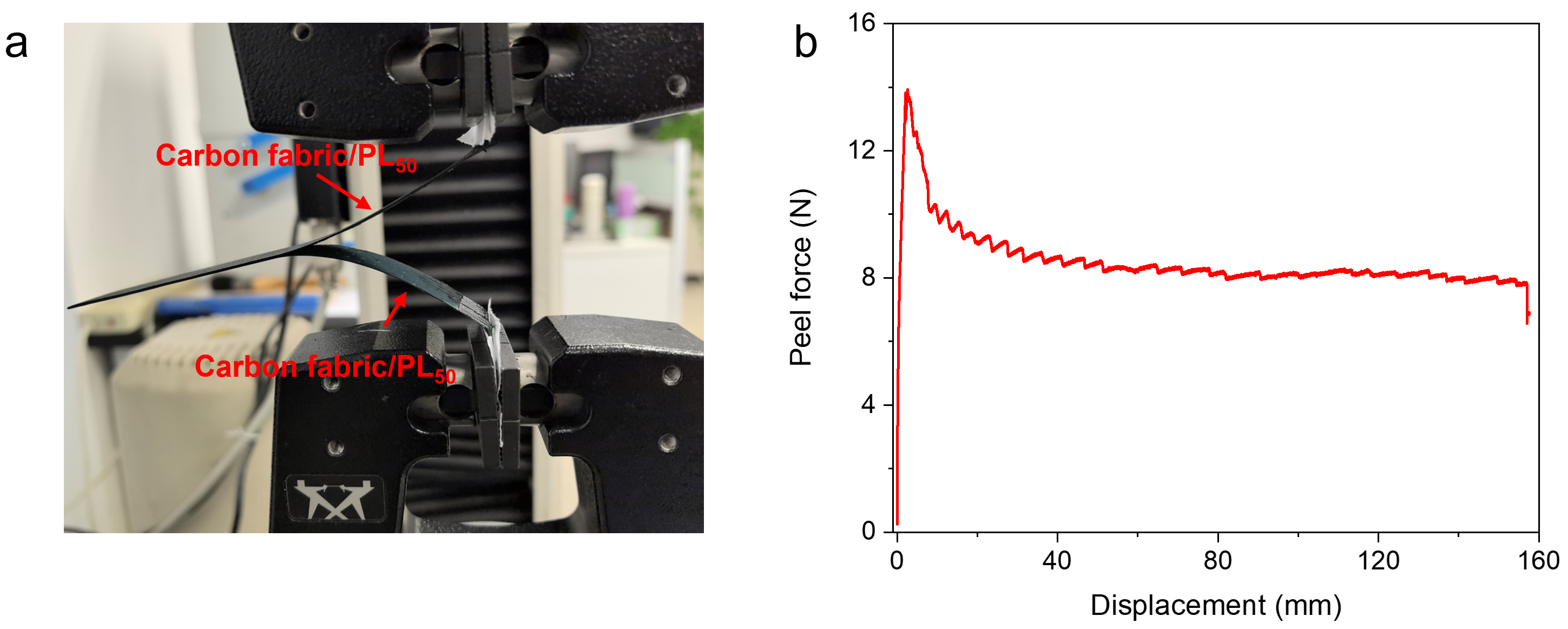


**Figure S24.** (a) Photograph of T-peel strength test setup. The dimensions of PL_50_@SBICs are 20 mm*120 mm and loading rate is 10 mm/min. (b) Peel force and displacement of PL_50_@SBICs.


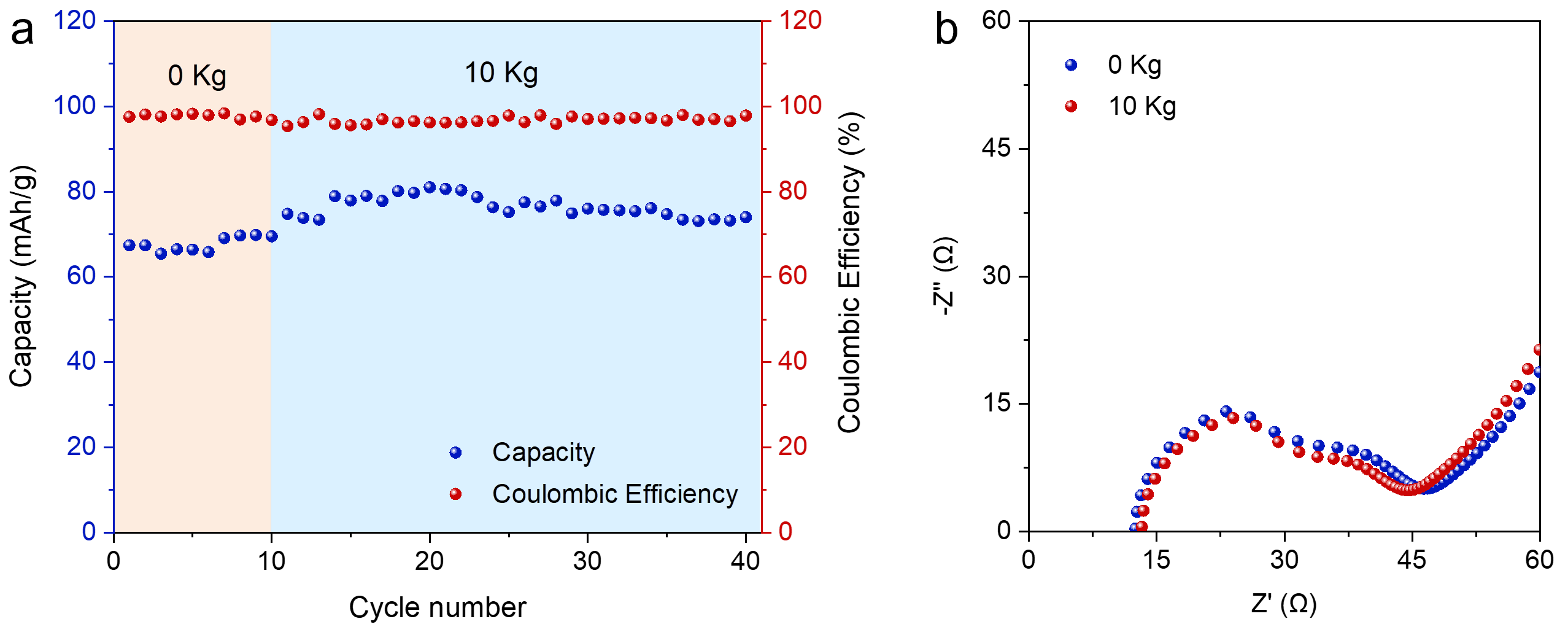


**Figure S25.** (a) Cycling performance of PL_50_@SBICs before and after supporting more than 10 kg of weight at a rate of 0.2 C, cycled at 25°C. (b) The EIS of PL_50_@SBICs before and after supporting more than 10 kg of weight.

The PL_50_@SBICs exhibited excellent mechanical property, with no significant changes in the coulombic efficiency after applying a weight of 10 kg. The slight increase in the specific capacity observed after applying pressure can be attributed to the enhanced contact between the electrodes, which reduces the interface impedance.


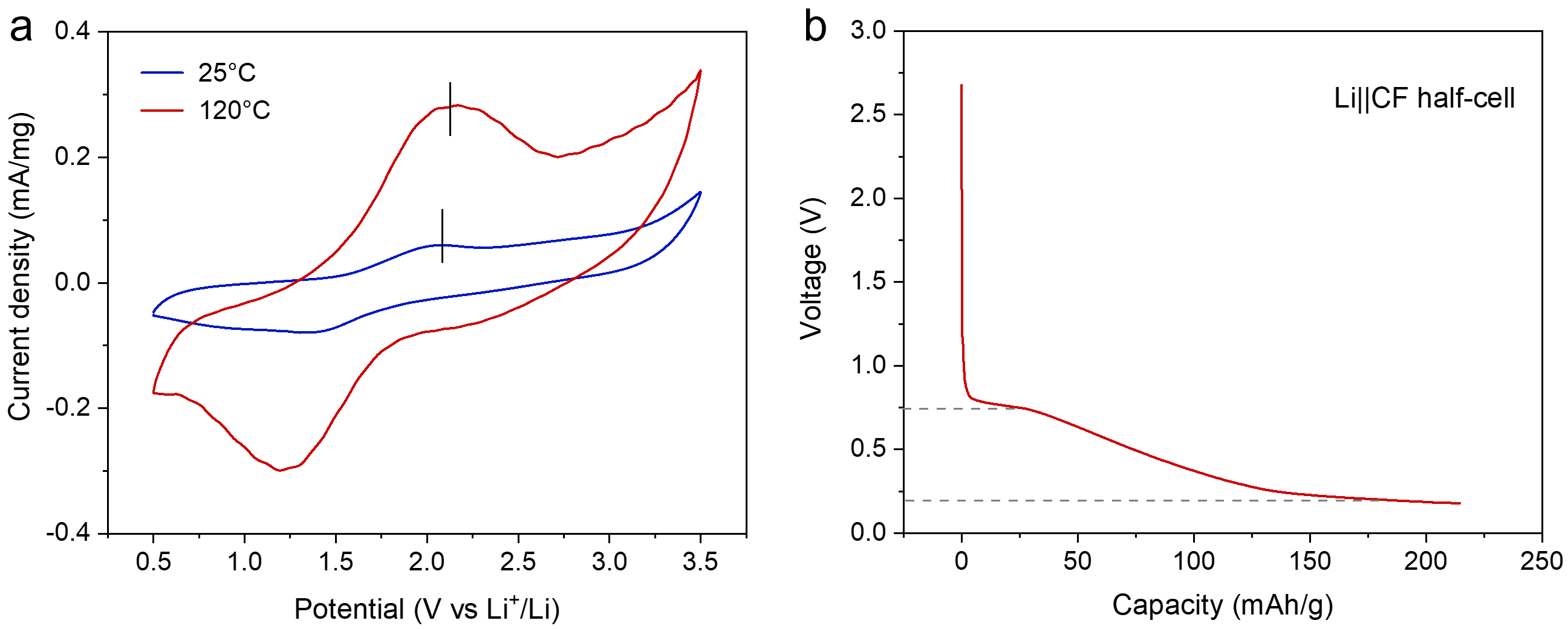


**Figure S26.** (a) Cyclic voltammetry curves of PL_50_@SBICs at 25°C and 120°C. (b) The discharge curves of Li||CF half-cell at a rate of 0.1 C.

The output voltage of the battery is determined by various factors such as the electrode potentials of the cathode and anode, as well as the impedance of the electrolyte. In PL_50_@SBICs, the cathode consists of LiFePO_4_ loaded onto carbon fibers, while the anode is composed of carbon fibers. Understanding the lithiation process of carbon fibers on the anode side is crucial in determining the output voltage of PL_50_@SBICs. Therefore, we assembled a Li||CF half-cell and conducted charge-discharge tests on it. As show in Figure S6, two lithiation plateaus are observed for carbon fibers at 0.7 V and 0.2 V, respectively. And the loading of LiFePO_4_ is 2 mg/cm^2^, and the surface density of carbon fibers is 30.7 mg/cm^2^. Consequently, SBIC's N/P ratio is approximately 20; so only the 0.7 V platform of the carbon fiber will be used. Furthermore, due to limitations in ion transport properties and thickness imposed by PL_50_ electrolyte, the lithiation potential of anode in the PL_50_@SBICs is further increased, resulting in a large difference between the redox potential of the battery and the redox potential of LiFePO_4_ (3.4-3.5 V vs. Li^+^/Li).


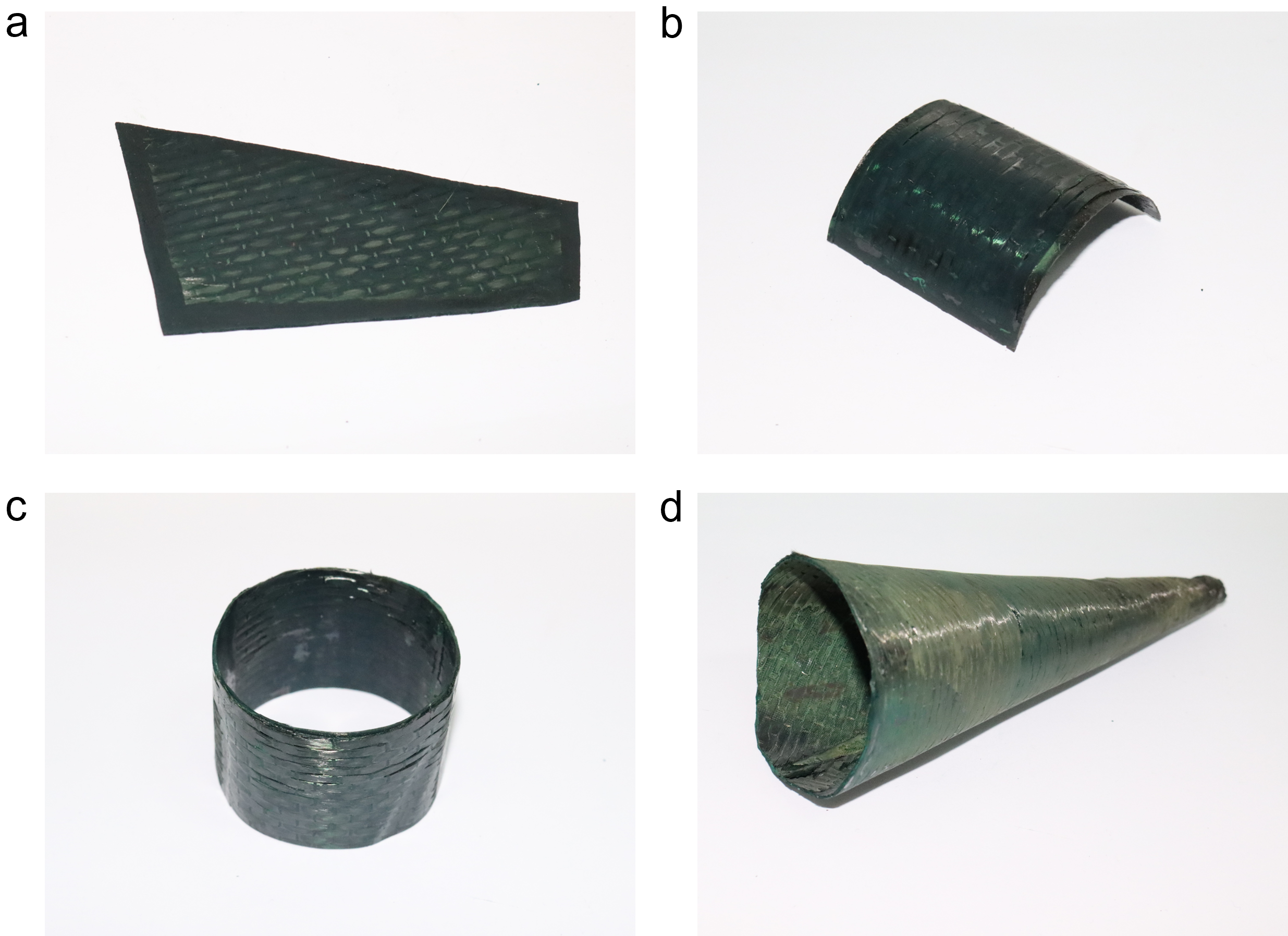


**Figure S27.** Photographs of PL_50_@SBICs with different shapes.


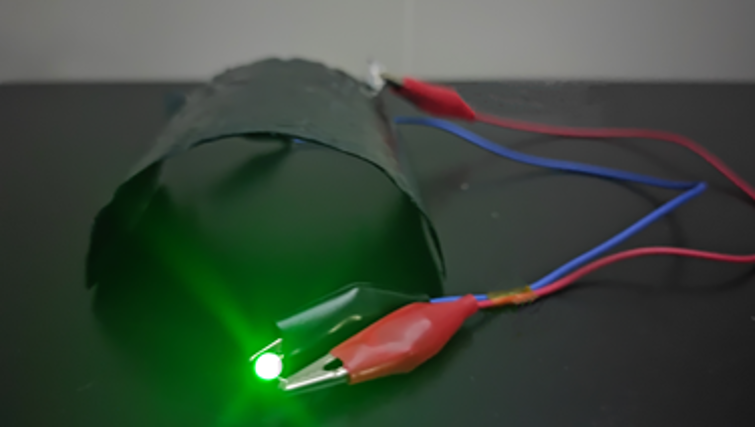


**Figure S28.** Photograph of the molded PL_50_@SBICs powering LEDs.


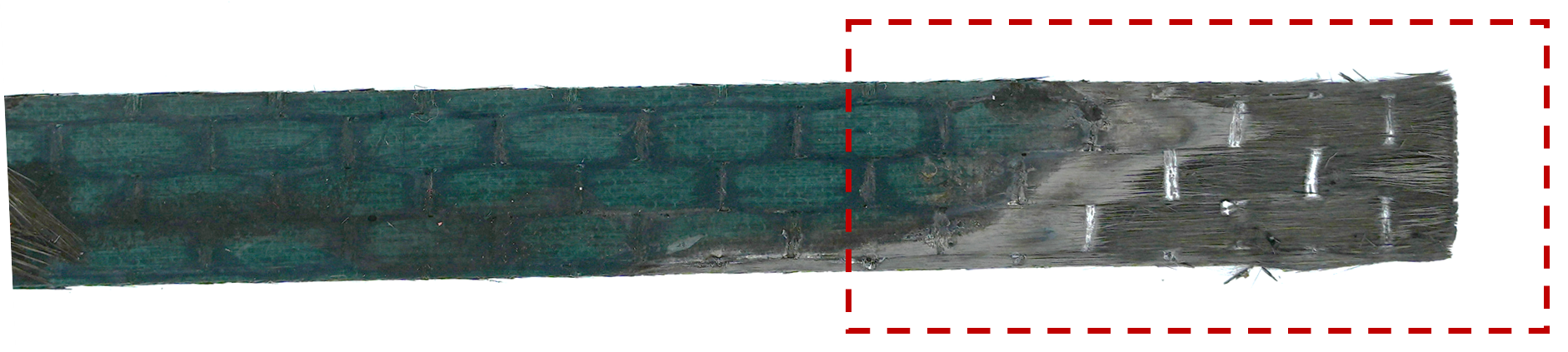


**Figure S29.** Photographs of the PL_50_@SBICs sample after the limit oxygen index test.

The carbon fiber electrodes remain well-preserved during combustion, thereby significantly improving the LOI of PL_50_@SBIC and providing enhanced safety for practical applications.

Table S1. Pore structure characteristic parameters of different samples by MIP.

| **Samples** | **Porosity**  **(%)** | **V_total_**  **(cm^3^/g)** | **Median Pore Diameter (Volumn)**  **(nm)** |
| --- | --- | --- | --- |
| PL_70_ | 23.1254 | 0.2527 | 9.05 |
| PL_60_ | 30.6784 | 0.3493 | 26.96 |
| PL_50_ | 38.7972 | 0.5194 | 229.71 |
| PL_40_ | 53.0344 | 0.9485 | 1992.58 |

Table S2. Pore structure characteristic parameters of PL_50_ samples containing different LiTFSI concentrations by MIP.

| **Samples** | **Porosity**  **(%)** | **V_total_**  **(cm^3^/g)** | **Median Pore Diameter (Volumn)**  **(nm)** |
| --- | --- | --- | --- |
| 0.5M | 39.1594 | 0.5573 | 421.15 |
| 1.0M | 44.4280 | 0.6269 | 380.47 |
| 2.0M | 36.5972 | 0.4781 | 250.75 |
| 2.3M | 38.7972 | 0.5194 | 229.71 |
| 3.0M | 36.3054 | 0.4945 | 168.49 |
| 4.0M | 39.3892 | 0.5187 | 119.10 |

Table S3. The impedance values of the equivalent circuit in Figure 4e.

| **Samples** | **R_b_(Ω)** | **R_SEI_(Ω)** | **R_ct_(Ω)** |
| --- | --- | --- | --- |
| 25°C | 14.10 | 12.03 | 59.15 |
| 120°C | 13.02 | 7.67 | 32.30 |

Table S4. Flame retardancy parameters obtained from the LOI and UL-94 tests.

| **Samples** | **LOI(%)** | **UL-94** | **Dripping** |
| --- | --- | --- | --- |
| PN | 32.2±0.4 | V-0 | No |
| PL_50_ | 32.1±0.4 | V-0 | Yes (IL) |
| PL_50_@SBIC | 50.7±0.1 | / | / |

In the UL-94 test, the drop of PL_50_ is ionic liquid electrolyte (ILE) that oozes from the surface of the sample and does not ignite the cotton on the lower side.

**References**

[1] D. Augustine, D. Mathew, C. P. R. Nair. *Polym. Int.* **2013**, *62*, 1068.

[2] K. Cheng, J. B. Lv, J. Z. Ma, J. H. Hu, C. Chen, K. Zeng, G. Yang*. Express Polym. Lett.* **2017**, *11*, 924.

[3] N. Shirshova, A. Bismarck, E. S. Greenhalgh, P. Johansson, G. Kalinka, M. J. Marczewski, M. S. P. Shaffer, M. Wienrich, *J. Phys. Chem. C* **2014**, *118*, 28377.
